# Supplementary material for: Validation of oxygen saturations measured in the community by emergency medical services as a marker of clinical deterioration in patients with confirmed COVID-19: a retrospective cohort study
Source: BMJ Open. 2024 Jan 2;14(1):e067378. doi: 10.1136/bmjopen-2022-067378 (PMC10773313; doi:10.1136/bmjopen-2022-067378)
Supplement: Author's manuscript [file bmjopen-2022-067378.draft_revisions.pdf]

BMJ Open is committed to open peer review. As part of this commitment we make the peer review history of every article we publish publicly available.

When an article is published we post the peer reviewers' comments and the authors' responses online. We also post the versions of the paper that were used during peer review. These are the versions that the peer review comments apply to.

The versions of the paper that follow are the versions that were submitted during the peer review process. They are not the versions of record or the final published versions. They should not be cited or distributed as the published version of this manuscript.

BMJ Open is an open access journal and the full, final, typeset and author-corrected version of record of the manuscript is available on our site with no access controls, subscription charges or pay-per-view fees (http://bmjopen.bmj.com).

If you have any questions on BMJ Open's open peer review process please email info.bmjopen@bmj.com

BMJ Open

Validation of oxygen saturations measured in the community by emergency medical services as a marker of clinical deterioration in patients with confirmed COVID-19

Journal:	*BMJ Open*
Manuscript ID	bmjopen-2022-067378
Article Type:	Original research
Date Submitted by the Author:	11-Aug-2022
Complete List of Authors:	Inada-Kim, Matthew; Hampshire Hospitals NHS Foundation Trust, Department of Acute Medicine<br>Chmiel, Francis P.; University of Southampton<br>Boniface, Michael; University of Southampton<br>Burns, Daniel; University of Southampton<br>Pocock, Helen ; South Central Ambulance Service NHS Foundation Trust; University of Warwick<br>Black, John; South Central Ambulance Service NHS Foundation Trust; Oxford University Hospitals NHS Foundation Trust, Emergency Department<br>Deakin, Charles ; South Central Ambulance Service NHS Foundation Trust; University Hospital Southampton NHS Foundation Trust
Keywords:	COVID-19, RESPIRATORY MEDICINE (see Thoracic Medicine), Epidemiology < INFECTIOUS DISEASES

SCHOLARONE™
Manuscripts

I, the Submitting Author has the right to grant and does grant on behalf of all authors of the Work (as defined in the below author licence), an exclusive licence and/or a non-exclusive licence for contributions from authors who are: i) UK Crown employees; ii) where BMJ has agreed a CC-BY licence shall apply, and/or iii) in accordance with the terms applicable for US Federal Government officers or employees acting as part of their official duties; on a worldwide, perpetual, irrevocable, royalty-free basis to BMJ Publishing Group Ltd ("BMJ") its licensees and where the relevant Journal is co-owned by BMJ to the co-owners of the Journal, to publish the Work in this journal and any other BMJ products and to exploit all rights, as set out in our licence.

The Submitting Author accepts and understands that any supply made under these terms is made by BMJ to the Submitting Author unless you are acting as an employee on behalf of your employer or a postgraduate student of an affiliated institution which is paying any applicable article publishing charge ("APC") for Open Access articles. Where the Submitting Author wishes to make the Work available on an Open Access basis (and intends to pay the relevant APC), the terms of reuse of such Open Access shall be governed by a Creative Commons licence – details of these licences and which Creative Commons licence will apply to this Work are set out in our licence referred to above.

Other than as permitted in any relevant BMJ Author's Self Archiving Policies, I confirm this Work has not been accepted for publication elsewhere, is not being considered for publication elsewhere and does not duplicate material already published. I confirm all authors consent to publication of this Work and authorise the granting of this licence.

For peer review only

Validation of oxygen saturations measured in the community by emergency medical services as a marker of clinical deterioration in patients with confirmed COVID-19

Matthew Inada-Kim[1]

Francis P. Chmiel[2]

Michael J. Boniface[2]

Helen Pocock[3,4]

John J. M. Black[3,5]

Charles D. Deakin[3,6]

[1] Acute Medical Unit, Department of Acute Medicine, Hampshire Hospitals NHS Foundation Trust, Winchester, United Kingdom.

[2] School of Electronics and Computer Science, University of Southampton, Southampton, SO17 1BJ, UK

[3] South Central Ambulance Service NHS Foundation Trust, Otterbourne, SO21 2RU, UK

[4] Warwick Clinical Trials Unit, University of Warwick, Coventry, CV4 7AL, UK

[5] Emergency Department, Oxford University Hospitals NHS Foundation Trust OX3 9DU.

[6] Southampton Respiratory Biomedical Research Unit, National Institute for Health Research, University Hospital Southampton, SO16 6YD

For peer review only

ABSTRACT

Background The early identification of deterioration in COVID-19 patients managed at home enables a more timely clinical intervention, which is likely to translate into improved outcomes. We undertook an analysis of COVID-19 patients conveyed by emergency medical services (EMS) to hospital to investigate how oxygen saturation and measurements of other vital signs correlate to patient outcomes, to ascertain if clinical deterioration can be predicted with simple community physiological monitoring.

Methods A retrospective analysis of routinely collected clinical data relating to patients conveyed to hospital by EMS was undertaken. We used descriptive statistics and predictive analytics to investigate how vital signs, measured in the community by EMS staff, correlate with patient outcomes. The primary study outcome was admission to ICU within 30-days of conveyance. ROC analysis was performed to evaluate, in a retrospective fashion, the efficacy of different variables in predicting patient outcomes.

Results We identified 1,080 adults with a COVID-19 diagnosis who were conveyed by EMS to either Basingstoke & North Hampshire Hospital or the Royal Hampshire County Hospital (Winchester) between March 1st and July 31st. Vital signs measured by EMS staff at first point of contact in the community correlated with patient 30-day ICU admission and mortality. Oxygen saturations were the most predictive of 30-day ICU admission (AUROC 0.753 (95 % CI: 0.668-0.826)), followed by the NEWS2 score (AUROC 0.731 (95 % CI: 0.655-0.800), temperature (AUROC 0.720 (95 % CI: 0.640-0.793)), and respiration rate (AUROC 0.672 (95 % CI: 0.586-0.756)).

Conclusions Initial oxygen saturation measurements (on air) for confirmed COVID-19 patients conveyed by EMS correlated with short-term patient outcomes, demonstrating an AUROC of 0.753 (95% CI: 0.668-0.826) in predicting 30-day ICU admission. We found that even small deflections in oxygen saturations confer an increased risk of adverse outcome in those with confirmed COVID at their initial community assessments.

For peer review only - http://bmjopen.bmj.com/site/about/guidelines.xhtml

KEY MESSAGES

What is already known on this subject

COVID-19 has high morbidity and mortality and earlier recognition enabling timely hospital admission, particularly in the light of recent discoveries of effective disease modifying treatments, is very likely to improve outcomes. Complex scoring system tools have been proposed to predict those at highest risk of deterioration but these are not always practical in community settings. Home self-monitoring of COVID-19 patients using pulse oximetry to detect early desaturation and enable timely hospital care is unproven but may have potential to improve mortality rates and a range of other clinical outcomes.

What this study adds

This is the first study to report that baseline (community) oxygen saturation measurements (on air) for confirmed COVID-19 patients conveyed by EMS to hospital for further management correlated with short-term (30-day) ICU admission and/or mortality. Oxygen saturations alone correlate with the need for ICU admission and early death. Small deflections in oxygen saturations below 96% (i.e. low normal range in adults) also confers an increased deterioration risk in patients with confirmed COVID-19 at the time of their initial community assessment by EMS.

For peer review only

For peer review only - http://bmjopen.bmj.com/site/about/guidelines.xhtml

INTRODUCTION

COVID-19 presents the biggest global healthcare challenge of our generation. As of February 2021, COVID-19 associated mortality stands at over 110,000 in the UK [1].COVID-19 presents a number of challenges in identifying optimal management pathways, not only in terms of the clinical care itself, but also identifying the stage at which hospital admission is necessary. Traditional management pathways involving paramedic assessment and conveyance to the Emergency Department (ED) for further review have proven impractical, not only because of the large numbers of patients involved, but because of the need to minimise contact of COVID-19 patients with others. Most patients who become symptomatic do so in a home environment where the majority will remain. In terms of optimising outcome, there is a need to understand which symptoms and signs in this environment are prognostic indicators of potential deterioration. The national recommendation for the implementation of COVID virtual wards recently announced by NHS England,[2] ushers in a novel approach of empowering patients through providing symptomatic, at risk patients a pulse oximeter and a toolkit for self-monitoring at home. It is hoped that this will enable the earlier recognition of deterioration in COVID-19 patients and potentially improved outcomes.

In most cases of bacterial and non-COVID pneumonia, breathlessness appears relatively early in the disease and ahead of any significant hypoxia. The challenge with assessing COVID-19 severity is that asymptomatic hypoxia often precedes breathlessness and by the time symptoms of breathlessness occur, patients have developed advanced disease and hypoxia may be significant.[3] The ability to detect this asymptomatic hypoxia before patients experience shortness of breath is critical for preventing respiratory involvement progressing to a life-threatening state. The key is to be able to detect this initial drop in oxygen saturation levels so that patients infected with COVID-19 who begin to suffer from pulmonary complications in the community can be detected early and conveyed to hospital for further treatment.[4] Although some studies have reported the relationship between oxygen saturation and outcome on presentation to the ED, we are not aware of any studies that have reported the relationship between oxygen saturations measured in the community by EMS and outcome. Patients who on assessment are severely hypoxic are clearly in need of emergency conveyance and hospital treatment, but by far the majority of patients with Covid-like symptoms seen and assessed by the EMS have relatively normal or near-normal oxygen saturations. These patients have generally not been conveyed and have been managed at home, but it has become apparent that even relatively minor derangements in oxygen saturations may be an early warning indicator for disease progression and the subsequent need for critical care. Use of oxygen saturation as an indicator of disease severity may therefore underestimate the risk of leaving patients at home after assessment by the EMS. National case fatality rates (CFR) (ratio of deaths to total cases) have

For peer review only - http://bmjopen.bmj.com/site/about/guidelines.xhtml

shown a strong inverse correlation between target oxygen saturation levels of 90-98% [5] suggesting that even mild derangements in oxygen saturation untreated can be detrimental to outcome.

Understanding the prognostic implications of oxygen saturation when first measured by EMS clinicians would enable safe and effective triage and potentially improve outcome through early identification of those most at risk of disease progression. Two small studies have suggested the utility of home oxygen monitoring for COVID-19 patients discharged from hospital,[6, 7] but no studies to our knowledge have used out-of-hospital oxygen saturation measurements as a trigger for initial hospital assessment. With second waves of COVID-19 sweeping most European countries, there is an urgent need to establish the prognostic significance of initial oxygen saturation to enable effective triage and optimise the use of limited healthcare resources, not only for those with COVID-19, but for the far greater majority with non-COVID-19 illness who have been deprived of timely healthcare as a consequence.

We therefore undertook a retrospective review of clinically confirmed COVID-19 patients accessing a regional UK ambulance service who were conveyed to hospital and correlated their initial oxygen saturations measured at home with their in-hospital outcome. These were compared with the standard NEWS2 patient score, as used by all UK ambulance services, to identify the deteriorating patient.[8]

For peer review only - http://bmjopen.bmj.com/site/about/guidelines.xhtml

METHODS

Study Design

We conducted a retrospective cohort analysis of adult patients (aged 18 years of older) initially assessed and conveyed by personnel from South Central Ambulance Service (SCAS) to the Emergency Department at one of the two hospitals within north Hampshire; Basingstoke & North Hampshire Hospital, or the Royal Hampshire County Hospital (Winchester) at which the patients were subsequently admitted

All calls to the relevant EMS, both emergency (999) and urgent (111) are triaged using NHS Pathways telephone script (release 19). We analysed EMS conveyances occurring between 1st March to 31st July 2020, to determine suspect COVID-19 among conveyances at initial time of contact by the call taker or EMS staff, each patient record was reviewed for inclusion of at least one of the following four identifiers:

1. Those in who the EMS call taker had classified the call as 'COVID– Respiratory Distress'
2. Those where the Patient Clinical Record (PCR) listed the 'Presenting complaint' as 'Suspected COVID-19'.
3. Those where the PCR free text for the 'Presenting complaint' contained the word 'COVID'
4. Those where the PCR narrative in the free text field summarising the symptoms and their details completed by the paramedic contained the word 'COVID'.

Conveyances from these suspect COVID-19 patients were then linked to their subsequent hospital attendance. Of suspect cases, we then identified confirmed COVID-19 cases by selecting only those with a confirmed diagnosis in their discharge summary (i.e., the presence of a U07.1 or U07.2 ICD10 code). These confirmed COVID-19 cases made up our study cohort.

All patients in known palliative care pathways were excluded from data analysis because their care did not follow standard care pathways.

Study setting

SCAS is a provider of emergency care in the counties of Hampshire, Berkshire, Buckinghamshire and Oxfordshire and covers a total of 3554 sq. miles (9205 km^2). The service receives approximately 500,000 emergency and urgent calls annually. SCAS covers a residential population of approximately 4.0 million inhabitants in a mix of urban and rural areas. The north Hampshire region forms part of the area covered by SCAS and comprises a residential population of approximately 306,000.[9]

For peer review only - http://bmjopen.bmj.com/site/about/guidelines.xhtml

Data collection

The initial oxygen saturation reading (SpO_2) on air recorded by the attending EMS staff (prior to any exercise or step test) and the NEWS2 score of patients fulfilling the inclusion criteria were collected from the EMS PCR. (NEWS2 score is calculated using the following seven variables: systolic blood pressure, heart rate, respiratory rate, temperature, oxygen saturation, supplemental oxygen administration, and level of consciousness - https://www.england.nhs.uk/ourwork/clinical-policy/sepsis/nationalearlywarningscore.)

Patient outcome was obtained by linking the SCAS and hospital clinical records by their NHS number. The primary outcome of our study was ICU admission within 30-days of conveyance and the secondary outcomes was mortality and a combined outcome (ICU admission and/or mortality) within 30-days of conveyance.

Data analysis

Analysis was performed in Python 3.7.2 [10], primarily making use of the statsmodels library [11]. Confidence intervals on observed mortality rates were estimated using the Wilson score interval. Where relevant, significance of the difference between two observed adverse outcome rates were tested using a two-population proportions z-test with the null hypothesis that the two-population proportions are equal.

To evaluate how predictive individual variables (e.g., oxygen saturation) and combinations of variables (e.g., oxygen saturation with age) were of 30-day adverse outcomes, we performed Receiving Operator Characteristics curve analysis (Table 2 and Table 3). In the univariate analysis, we performed a complete case analysis (removing any patient with an incomplete record of vital signs, Table 1) and assume a patient's adverse outcome risk is a linear function of the respective variable (where negative or positive correlation with outcome is assessed by clinical judgement) and calculated the ROC curve corresponding to if this variable alone was used to predict a patients risk of an adverse outcome. We present both the sensitivity and specificity or the Area Under the Receiving Operator Characteristic curve (AUROC). The AUROC provides an estimate of the degree to which the predictor can discern between whether a patient has an adverse outcome within 30 days of conveyance or not, it can take values between 0.5 and 1.0. An AUROC of 0.5 corresponds to randomly guessing which patient have an adverse outcome within 30 days and an AUROC of 1.0 corresponds to a perfect classifier - it can predict, without error, who will have an adverse outcome within 30-days of conveyance. Confidence intervals were estimated by performing 1000

For peer review only

For peer review only - http://bmjopen.bmj.com/site/about/guidelines.xhtml

bootstrapping (sampling with replacement) iterations on the available data, calculating the AUROC on each of the samples and then calculating the relevant percentiles.

Patient and Public Involvement

This research was done without patient involvement. Patients were not invited to comment on the study design and were not consulted to develop patient-relevant outcomes or interpret the results. Patients were not invited to contribute to the writing or editing of this document for readability or accuracy.

Governance and ethics approval

Regulatory and ethical approval for the study were provided by the Health Research Authority (REC reference 20/HRA/5445) and by the University of Southampton Ethics Committee (REF ERGO/61242). NHS England and NHS Improvement have been given legal notice by the Secretary of State for Health and Social Care to support the processing and sharing of information to help the COVID-19 response under Health Service Control of Patient Information Regulations 2002 (COPI). This is to ensure that confidential patient information can be used and shared appropriately and lawfully for purposes related to the COVID-19 response. Data were extracted from medical records by clinicians providing care for the patients and an anonymised extract of the data were provided to the team at the University of Southampton.

For peer review only

For peer review only - http://bmjopen.bmj.com/site/about/guidelines.xhtml

RESULTS

A total of 19,868 patients were assessed at home and subsequently conveyed by EMS to North Hampshire Hospitals during the study period. The call handler or EMS staff identified 2,257 suspect COVID-19 cases and of these we identified 1,209 adults as having a confirmed diagnosis of COVID-19 (U07.1 or U07.2 coded in the patients discharge summary). Of the 1,209 confirmed cases we removed persons under palliative care (112 patients) and those with no initial oxygen saturation measurement on air recorded (17 patients). Overall, this left us with 1,080 confirmed COVID-19 patient records all of whom had initial oxygen saturation measurements on air. Of these 1,080, the complete records of vital signs were recorded at home by paramedics for 892 of the patients (Table 1). In our following discussions, we make use of all 1,080 patients, with the exception for our univariate analysis (discussed in Table 3) where we perform a complete case analysis and only use the 892 complete records.

Oxygen saturation was found to correlate with adverse outcome (Figure 2), with lower initial oxygen saturation readings being associated with a higher mortality rate. In Figure 2 we display the correlation between the observed 30-day adverse outcome rates and initial oxygen saturation in detail, which displays correlation to all outcomes. In Table 2 we display the breakdown of our retrospective ROC analysis for using measured oxygen saturation as a binary triage tool (i.e., hospitalize or not) for different cut-offs (rows in Table 2). While the sensitivity vs specificity trade-off needs to be determined by the clinical context, this demonstrates that oxygen saturation is moderately discriminative for several cut-offs. For example, for a cut-off of 94 % or below, the sensitivity is 0.742 (95 % CI: 0.642-0.734) and the specificity is 0.706 (95 % CI: 0.678-0.734). Finally, we present comparisons of the results of ROC analysis for different variables measured in the community by EMS (Table 3). Across the three presented outcomes (30-day ICU admission, mortality, and combined outcome) correlations between variables and outcomes are broadly similar, with measured oxygen saturations and the NEWS2 score being the two most predictive of outcome. The notable differences are for the measured temperature which is moderately predictive of ICU admission (AUROC: 0.720 (95 % CI: 0.640-0.793)) but only weakly predictive of mortality (AUROC: 0.597 (95 % CI: 0.523-0.678)) and for patient age which is strongly positively correlated to mortality but displays a negative correlation to ICU admission (Inspect Figure 1 a and b).

For peer review only

Vital sign	Number missing	Percent missing
**Heart rate**	10	0.9
**Systolic blood pressure**	100	9.3
**Respiration rate**	120	11.1
**Oxygen Saturation (on air)**	0	0
**Temperature**	150	13.9
**ACVPU**	125	11.6
**Complete Records**	**892**	**83.0**

Table 1: Number of vital sign measurements missing and the number of complete records from 1,080 patient records. ACVPU = alert, confused, responding to voice, responding to pain, unresponsive. Oxygen saturations were not missing for any patients as those with missing values had been excluded (n=17). Overall, records were complete for 83% of cases.

For peer review only

For peer review only - http://bmjopen.bmj.com/site/about/guidelines.xhtml

Oxygen Saturation (on air) threshold (%)	Sensitivity (95 % CI)	Specificity (95 % CI)	Number of observations	Cumulative sum of number of observations
**85**	0.294 (0.200-0.400)	0.947 (0.933-0.962)	8	76
**86**	0.316 (0.216-0.421)	0.941 (0.927-0.955)	8	84
**87**	0.320 (0.216-0.432)	0.935 (0.920-0.950)	6	90
**88**	0.370 (0.261-0.476)	0.916 (0.899-0.933)	23	113
**89**	0.413 (0.304-0.523)	0.894 (0.874-0.913)	25	138
**90**	0.512 (0.411-0.615)	0.870 (0.849-0.890)	32	170
**91**	0.590 (0.477-0.699)	0.841 (0.823-0.867)	31	201
**92**	0.655 (0.544-0.761)	0.817 (0.796-0.841)	33	234
**93**	0.706 (0.593-0.803)	0.776 (0.751-0.801)	45	279
**94**	0.742 (0.642-0.840)	0.706 (0.678-0.734)	74	353
**95**	0.808 (0.718-0.892)	0.634 (0.605-0.662)	76	429
**96**	0.848 (0.767-0.921)	0.508 (0.477-0.538)	129	558
**97**	0.898 (0.822-0.963)	0.357 (0.330-0.386)	156	714
**98**	0.911 (0.841-0.973)	0.226 (0.201-0.254)	132	846
**99**	0.961 (0.913-1.0)	0.091 (0.075-0.109)	139	985
**100**	1	0	95	1080

Table 2: Evaluation of initial oxygen saturation measured by paramedics in COVID-19 patients in the community used as a binary classifier for predicting 30-day ICU admission within 30 days of conveyance. Each row denotes a different threshold for determining those at risk of an adverse outcome. We display the sensitivity and specificity for each threshold, equivalent to all possible intersections of the receiving operator curve using thresholds between 85 % and 100 %. In total 68 patients had an oxygen saturation of 84 % or less (not shown). The column on the far right denotes the cumulative sum of the number of observations of the given oxygen saturation (row) or below. For example, 76 patients had an oxygen saturation of 85 % or less recorded (top row) and 429 patients had an oxygen saturation of 95 % or less recorded. Confidence intervals are estimated by bootstrapping.

For peer review only

For peer review only - http://bmjopen.bmj.com/site/about/guidelines.xhtml

	AUROC (95 % CI)		
Variable	ICU admission	Mortality	Combined
**Oxygen Saturation (on air)**	0.753 (0.668-0.826)	0.778 (0.704-0.843)	0.775 (0.727-0.829)
**NEWS2**	0.731 (0.655-0.800)	0.768 (0.709-0.823)	0.760 (0.708-0.807)
**Respiration rate**	0.672 (0.586-0.756)	0.668 (0.599-0.736)	0.677 (0.618-0.738)
**Temperature**	0.720 (0.640-0.793)	0.597 (0.523-0.678)	0.636 (0.69-0.700)
**Systolic blood pressure**	0.634 (0.560-0.706)	0.604 (0.529-0.680)	0.626 (0.568-0.684)
**Heart rate**	0.590 (0.506-0.672)	0.558 (0.486-0.631)	0.574 (0.514-0.633)
**Age band**	0.670 (0.611-0.734)	0.685 (0.626-0.738)	0.557 (0.495-0.615)

Table 3: Ranked Area Under Receiver Operator Curves (AUROC) calculated for isolated physiological variables and the composite NEWS2 score with each outcome. AUROCS were calculated using a complete case analysis (see Table 1) with 892 patients in total. Confidence intervals are estimated by bootstrapping, with 95 % confidence intervals presented alongside the mean validation AUROC across samples.

For peer review only - http://bmjopen.bmj.com/site/about/guidelines.xhtml

DISCUSSION

Community assessment of patients with COVID-19 symptoms using a single initial oxygen saturation on air measurement correlates with 30-day clinical outcomes. Qualitatively, the observed 30-day adverse outcome rate is approximately constant between oxygen saturations of 100 - 96 % and then increase with decreasing oxygen saturation from 95 % to 90 %. Below 90 %, the mortality risk remains high. Although the therapeutic target range for oxygen saturations in the UK is 94-98%,[12] and in the USA is 92-96%,[13] this study suggests that patients at the lower end of this range are still at risk of deterioration in the context of COVID-like symptoms. For example, for patients in our cohort with presenting oxygen saturations in the range of 92-94 %, values often regarded as within this normal range, had a significantly (p=0.025) higher risk of ICU admission within 30 days (5.9 %) compared to those presenting with oxygen saturations greater than 95 % (ICU admission rate 2.5 %). Outside this 'normal' range, our analysis suggests even relatively small decreases in oxygen saturation are markers of increased risk of death or ICU admission and suggest that a lower threshold for hospital conveyance may be necessary for patients who traditionally would be considered to have only minor physiological derangement and otherwise have been left at home.

The sensitivity of home oxygen saturation measurements reflects the percentage of people correctly identified with adverse outcomes. The sensitivity of this parameter for adverse outcome decreased as oxygen saturation fell (Table 2). An oxygen saturation ≤ 90% was associated with a relatively low sensitivity of < 0.5. Specificity of identifying an adverse outcome, an indirect measure of unnecessary conveyance to hospital (but also including patients who survived and did not need ICU admissions), increased as oxygen saturations fell. However, it is important to ensure that patients at risk of deterioration are not missed and a degree of over-triage would be necessary to ensure that this was not the case. However, even oxygen saturations at the lower end of the normal range are associated with a risk of deterioration (sensitivity of 94% saturations = 0.713) and it therefore appears that oxygen saturation alone has significant limitations when it is within a normal range.

Although oxygen saturations as a risk factor for COVID-19 patients on presentation to the Emergency Department are widely reported,[14, 15,16] the ability of oxygen saturations measured in the community to indicate disease severity and the need for hospital conveyance has not been widely reported, presumably because of the challenges in equipping patients with pulse oximeters prior to the onset of any illness. Several studies have used oxygen levels in patients presenting in the ED as an indicator of the need for hospital admission and others have used the opportunity to send ED patients not requiring admission home with a pulse oximeter for self-monitoring. Oxygen saturations

For peer review only

on presentation to the ED have also been shown to be strongly associated with outcome. The strongest critical illness risk has been shown to be admission oxygen saturation < 88% (OR 6.99).[15] Other studies have shown that even a relatively mildly deranged oxygen saturation of <92% is strongly associated with an increased risk of in-hospital mortality.[17] Conversely, an ED resting SpO_2 ≥ 92% as part of discharge criteria can achieve hospital readmission rates as low as 4.6%, [16] suggesting that it may be a safe threshold for discharge in symptomatic patients with mild disease after diagnostic workup .

Home oxygen saturation monitoring has been used for patients discharged from hospital, either from the ED because their disease was not severe, or from intensive care for convalescence. A small study of patients with COVID-19 discharged from an ED, reported similar results to ours using subsequent home oxygen saturation monitoring. In these patients, resting home SpO_2 < 92% was associated with an increased likelihood of re-hospitalization compared to SpO_2 ≥ 92% (relative risk = 7.0, 95% CI 3.4 to 14.5, $p < 0.0001$). Home SpO_2 < 92% was also associated with increased risk of intensive care unit admission.[7]

Oxygen saturation is an integral variable in most critical illness tools that have been used to identify COVID-19 patients requiring hospital admission.[18] NHS England has encouraged the use of the NEWS2 scoring system to identify patients at risk of deterioration. This uses weighted physiological variables of heart rate, systolic blood pressure, oxygen saturation (on air), respiratory rate, temperature and level of consciousness to produce a score that is correlated with risk of deterioration, not only as a general illness score, but specifically in patients with known COVID-19.[19] We therefore compared the ability of isolated oxygen saturations with NEWS2 in our cohort to identify patients at risk of ICU admission (and mortality) within 30 days. Using ROC analysis, the AUROC for oxygen saturations at predicting ICU admission alone was 0.753 (95% CI 0.668-0.826) and for NEWS2 was 0.731 (95% CI 0.655-0.800). These results are consistent with a previous study using NEWS2 scores on hospital admission which has shown an AUROC of 0.822 (95% CI 0.690-0.953) to predict risk of severe disease.[19] The lower observed AUROC of NEWS2 compared to oxygen saturations may be the result of the NEWS2 score incorporating physiological variables less predictive of COVID-19 outcomes than oxygen saturations, thereby reducing the discriminative ability of the score, or because it uses discretized oxygen saturations which amounts to information loss. Additionally, we have not assessed the reporting compliance of the NEWS2 scores and this may have impacted the observed AUROCs. Interestingly, a recent review of 22 prognostic models showed that oxygen saturation on room air and patient age were strong predictors of deterioration and mortality among hospitalised adults with COVID-19 respectively, but no other variables added

For peer review only

For peer review only - http://bmjopen.bmj.com/site/about/guidelines.xhtml

incremental value to these predictors.[18] We have shown the same for oxygen saturation as a univariate predictor in the pre-hospital setting, and that predictive value does not increase by the addition of other physiological variables. The PRIEST study using NEWS2, age, sex, and performance status of patients in the ED predicted adverse outcome with good discrimination in adults with suspected COVID-19 [20]. The discriminatory ability of this more complex scoring system was similar to that demonstrated by simply measuring the oxygen saturations in the community and further reinforces the utility of home oxygen saturations as a simple marker, not only for use by the EMS, but by members of the public equipped with home oximetry.

A number of remote home monitoring models for patients with suspected COVID-19 have been proposed, all of which aim to achieve early identification of deterioration for patients self-managing COVID-19 symptoms at home.[21] It would be expected that the utility of home monitoring would be improved by the ability to measure oxygen saturations, although not all models currently integrate this into their protocols. Our results show that resting oxygen saturations measured in patients with confirmed COVID-19 perform on a par with the same measurements taken in the ED. They therefore suggest that the predictive value of oxygen saturations may be able to be effectively moved to an earlier stage in the disease process and measured while the patient is still at home. Although initial home SpO_2 may provide a useful marker of disease severity and the need for hospital conveyance, it is clear that it has limited sensitivity and may need to be interpreted as part of an overall assessment of the patient. Some authors have argued that pulse oximetry identified the need for hospitalisation when using a cut- off of 92%,[7] but based on our data (Table 2), approximately one-third of patients with an adverse outcome would be missed using this threshold. We have demonstrated that even patients presenting with oxygen saturations of 92-94 %, which are values often regarded as within a normal range, have a higher mortality than those with oxygen saturations higher than 95 %. Even when measured in the ED, baseline median SpO_2 was as high as 95.0 % in those with an adverse outcome, compared to 97.0% in those without.[22] It is clear that the relatively low sensitivity of oxygen saturation in those with mildly deranged values limits the utility of this parameter alone in assessing risk of adverse outcome.

This is a relatively small retrospective cohort study with concomitant limitations of sample size. The subjective nature of paramedic classification of symptoms consistent with COVID-19 may have introduced some degree of bias into patients included in the study, as may have the presence of known co-morbidities. Our dataset did not include patients who were reviewed by EMS but not conveyed to hospital and this is arguably the most significant source of bias in our study. It is

For peer review only - http://bmjopen.bmj.com/site/about/guidelines.xhtml

reasonable that for patients where a decision was made not to convey them, they were less likely to deteriorate and more likely to have normal vital signs. If this is the case, this would result in a reduction of the discriminative ability of recorded oxygen saturations. We did not specifically compare the outcome data of COVID and Non-COVID patients with mildly deranged oxygen saturations. However, our data suggests that mild derangement in COVID patients is a significant risk factor for deterioration and this does not match the clinical progression witnessed in non-COVID patients. Seventeen patients did not have initial oxygen saturations recorded on air (but did have oxygen saturations recorded on oxygen) and were excluded from the data analysis. If this was because they were so obviously hypoxic clinically that EMS staff immediately administered oxygen without an initial reading on air (or were constantly on home oxygen treatment), the ability of oxygen saturations to indicate risk of deterioration is likely to have been underestimated in this study. Patients on palliative care pathways were also removed from the study cohort, but are likely to be more susceptible to deterioration from COVID, irrespective of any alternative care pathway.

With waves of COVID-19 regularly overwhelming EMS and hospital services, there is an urgent need to optimise the identification of patients at risk of deterioration. We undertook this research to ascertain the role simple physiological measures might have to inform clinical decision making. While the results are hypothesis-forming (i.e., it shows oxygen saturations are predictive of clinical outcomes within the care pathway studied in this manuscript), it has clinical utility as it helps inform decisions made by clinicians at the point of conveyance. This will enable more patients to be safely managed in the community and only referred to hospital once their clinical symptoms and physiological signs suggest a risk of deterioration and the need for hospital care. This is particularly needed for the majority of patients who have mild to moderate symptoms where it is not clear if community or hospital management is appropriate. Home pulse oximetry is becoming relatively cheap and easily accessible for the public and may be a relatively cost-effective tool in the safe community management of these patients, perhaps focussed on those with significant co-morbidities who are at higher risk. The utility of remote monitoring systems (or the COVID virtual ward) has been an increasingly studied subject, and there is growing evidence that remote monitoring can facilitate more streamlined approaches to the delivery of patient care, especially in pulmonary disease.[6] The use of ICU admission as an endpoint identifies patients seen at home who go on to deteriorate and the correlation of home oxygen saturation with a risk of severe deterioration assists ambulance crews in identifying both those who should be conveyed to hospital as well as those who can, with a reasonable degree of certainty, be safely left at home. Further prospective studies are required to understand the utility of home pulse oximetry, but this study

For peer review only

For peer review only - http://bmjopen.bmj.com/site/about/guidelines.xhtml

suggests that it may have the potential to significantly contribute to the safe and appropriate management of these patients in the community with timely referral to hospital when indicated.

Conclusions

We have demonstrated that even relatively minor derangements in peripheral oxygen saturation are an early warning of potential deterioration in confirmed COVID-19 patients conveyed by EMS to hospital and oxygen saturation would appear to have potential to be a key physiological variable that together with other clinical signs and clinical risk factors may be able to identify patients at risk of deterioration.

Acknowledgements

We thank Simon Mortimore and Philip King from South Central Ambulance Service and Zoe Cameron from Hampshire Hospitals NHS Foundation Trust for their assistance in data extraction and analysis.

This report includes independent research funded by the National Institute for Health Research Applied Research Collaboration Wessex. The views expressed in this publication are those of the author(s) and not necessarily those of the National Institute for Health Research or the Department of Health and Social Care.

Competing interests

M. I-K. is National Clinical Lead Deterioration & National Specialist Advisor Sepsis, NHS England and NHS Improvement. All other authors declare no competing interests.

Data accessibility

Due to information governance concerns, the data will not be made public. However, it will be made accessible via reasonable request to the corresponding author.

For peer review only

References

1. England, P.H. *Coronavirus (COVID-19) in the UK.*; Available from: https://coronavirus.data.gov.uk.
2. *Pulse oximetry to detect early deterioration of patients with COVID-19 in primary and community care settings, 11 June 2020, Version 1, NHS.* https://www.england.nhs.uk/coronavirus/publication/pulse-oximetry-to-detect-early-deterioration-of-patients-with-covid-19-in-primary-and-community-care-settings *Accessed 28 Oct 2020.*
3. Bickler, P.E., et al., *"Silent" Presentation of Hypoxemia and Cardiorespiratory Compensation in COVID-19.* Anesthesiology, 2020.
4. Teo, J., *Early Detection of Silent Hypoxia in Covid-19 Pneumonia Using Smartphone Pulse Oximetry.* J Med Syst, 2020. **44**(8): p. 134.
5. Goyal, D., Donnelly H., Kussner, A., Bhatti, N.J., Mansab, F., *Oxygen and mortality in COVID-19 pneumonia: a comparative analysis of supplemental oxygen policies and health outcomes across 26 countries.* 2020.
6. O'Carroll, O., et al., *Remote monitoring of oxygen saturation in individuals with COVID-19 pneumonia.* Eur Respir J, 2020. **56**(2).
7. Shah, S., et al., *Novel use of home pulse oximetry monitoring in COVID-19 patients discharged from the emergency department identifies need for hospitalization.* Acad Emerg Med, 2020.
8. *Royal College of Physicians National Early Warning Score (NEWS) 2. London: RCP, 2017.* www.rcplondon.ac.uk/projects/outputs/national-early-warning-score-news-2 *[Accessed 19 Oct 2020].*
9. https://www.hants.gov.uk/landplanningandenvironment/facts-figures/population/estimates-forecasts. *Accessed 17th October 2020.*
10. *Python Software Foundation. Python Language Reference, version 3.7.2. Available at http://www.python.org*
11. *Seabold, S.*, Perktold, J. statsmodels: Econometric and statistical modeling with python. Proceedings of the 9th Python in Science Conference. 2010.
12. O'Driscoll, B.R., et al., *BTS guideline for oxygen use in adults in healthcare and emergency settings.* Thorax, 2017. **72**(Suppl 1): p. ii1-ii90.
13. *COVID-19 Treatment Guidelines Panel. Coronavirus Disease 2019 (COVID-19) Treatment Guidelines. National Institutes of Health. Available at* https://www.covid19treatmentguidelines.nih.gov/. *Accessed 15th October 2020.*
14. Gidari, A., et al., *Predictive value of National Early Warning Score 2 (NEWS2) for intensive care unit admission in patients with SARS-CoV-2 infection.* Infect Dis (Lond), 2020. **52**(10): p. 698-704.
15. Petrilli, C.M., et al., *Factors associated with hospital admission and critical illness among 5279 people with coronavirus disease 2019 in New York City: prospective cohort study.* BMJ, 2020. **369**: p. m1966.
16. Berdahl, C.T., et al., *The safety of home discharge for low-risk emergency department patients presenting with coronavirus-like symptoms during the COVID-19 pandemic: A retrospective cohort study.* JACEPOPEN, 2020. **August 2020.** https://doi.org/10.1002/emp2.12230.
17. Mikami, T., et al., *Risk Factors for Mortality in Patients with COVID-19 in New York City.* J Gen Intern Med, 2020.
18. Gupta, R.K., et al., *Systematic evaluation and external validation of 22 prognostic models among hospitalised adults with COVID-19: An observational cohort study.* Eur Respir J, 2020.

For peer review only

For peer review only - http://bmjopen.bmj.com/site/about/guidelines.xhtml

19. Myrstad, M., et al., *National Early Warning Score 2 (NEWS2) on admission predicts severe disease and in-hospital mortality from Covid-19 - a prospective cohort study.* Scand J Trauma Resusc Emerg Med, 2020. **28**(1): p. 66.

20. Goodacre S, et al. *Derivation and validation of a clinical severity score for acutely ill adults with suspected COVID-19: The PRIEST observational cohort study.* PLoS ONE 16(1), 2021

21. Vindrola-Padros, C., et al., *Remote home monitoring (virtual wards) during the COVID-19 pandemic: a living systematic review. 2020.* https://www.medrxiv.org/content/10.1101/2020.10.07.20208587v1.full.pdf.

22. *Goodacre, S. , Thomas, B., Lee, E. et al. Post-exertion oxygen saturation as a prognostic factor for adverse outcome in patients attending the emergency department with suspected COVID-19 : observational cohort study. medRxiv. (Submitted:2020).*

For peer review only

For peer review only

For peer review only - http://bmjopen.bmj.com/site/about/guidelines.xhtml

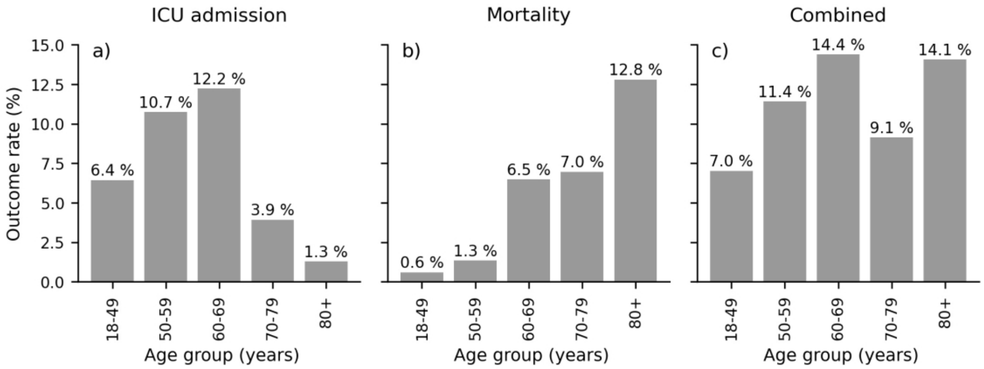

Figure 1: Observed a) ICU admission rate, b) 30-day mortality rate, and c) combined rates by age group for suspected COVID-19 patients conveyed by ambulance. Annotations (above bars) display the observed rates for the respective group.

184x70mm (300 x 300 DPI)

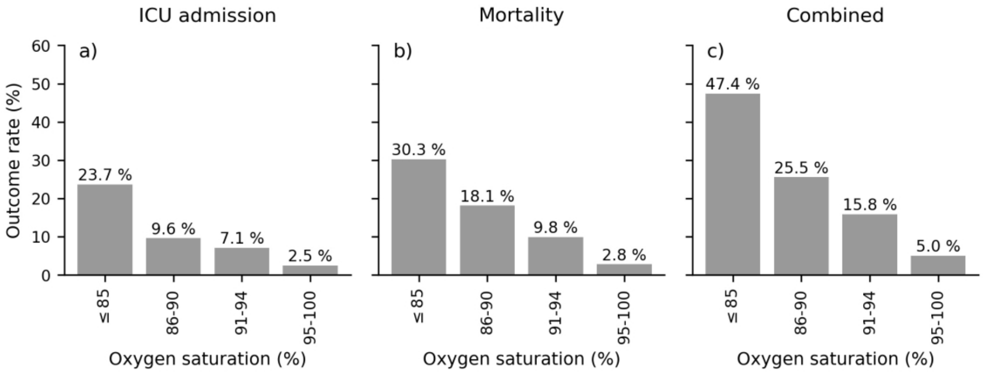

Figure 2: Observed a) ICU admission rate, b) 30-day mortality rate, and c) combined rates as a function of initial oxygen saturation (on air) measurement made by ambulance crews in the community. Annotations (above bars) display the observed rates for the respective group.

181x70mm (300 x 300 DPI)

STROBE Statement—Checklist of items that should be included in reports of ***cohort studies***

	Item No	Recommendation	Page No
**Title and abstract**	1	(*a*) Indicate the study's design with a commonly used term in the title or the abstract	1
		(*b*) Provide in the abstract an informative and balanced summary of what was done and what was found	2
**Introduction**			
Background/rationale	2	Explain the scientific background and rationale for the investigation being reported	5
Objectives	3	State specific objectives, including any prespecified hypotheses	6
**Methods**			
Study design	4	Present key elements of study design early in the paper	7
Setting	5	Describe the setting, locations, and relevant dates, including periods of recruitment, exposure, follow-up, and data collection	7, 8
Participants	6	(*a*) Give the eligibility criteria, and the sources and methods of selection of participants. Describe methods of follow-up	7, 8
		(*b*) For matched studies, give matching criteria and number of exposed and unexposed	
Variables	7	Clearly define all outcomes, exposures, predictors, potential confounders, and effect modifiers. Give diagnostic criteria, if applicable	8, 9
Data sources/ measurement	8*	For each variable of interest, give sources of data and details of methods of assessment (measurement). Describe comparability of assessment methods if there is more than one group	8, 9
Bias	9	Describe any efforts to address potential sources of bias	16
Study size	10	Explain how the study size was arrived at	N/A
Quantitative variables	11	Explain how quantitative variables were handled in the analyses. If applicable, describe which groupings were chosen and why	8, 9
Statistical methods	12	(*a*) Describe all statistical methods, including those used to control for confounding	8, 9, 10
		(*b*) Describe any methods used to examine subgroups and interactions	
		(*c*) Explain how missing data were addressed	
		(*d*) If applicable, explain how loss to follow-up was addressed	
		(*e*) Describe any sensitivity analyses	
**Results**			
Participants	13*	(a) Report numbers of individuals at each stage of study—eg numbers potentially eligible, examined for eligibility, confirmed eligible, included in the study, completing follow-up, and analysed	7, 10
		(b) Give reasons for non-participation at each stage	
		(c) Consider use of a flow diagram	
Descriptive data	14*	(a) Give characteristics of study participants (eg demographic, clinical, social) and information on exposures and potential confounders	Figures 1, 2
		(b) Indicate number of participants with missing data for each variable of interest	Pages 11, 12, 13
		(c) Summarise follow-up time (eg, average and total amount)	
Outcome data	15*	Report numbers of outcome events or summary measures over time	10

For peer review only - http://bmjopen.bmj.com/site/about/guidelines.xhtml

Main results	16	(*a*) Give unadjusted estimates and, if applicable, confounder-adjusted estimates and their precision (eg, 95% confidence interval). Make clear which confounders were adjusted for and why they were included<br>(*b*) Report category boundaries when continuous variables were categorized<br>(*c*) If relevant, consider translating estimates of relative risk into absolute risk for a meaningful time period	10
Other analyses	17	Report other analyses done—eg analyses of subgroups and interactions, and sensitivity analyses	11, 12, 13
**Discussion**			
Key results	18	Summarise key results with reference to study objectives	14, 15
Limitations	19	Discuss limitations of the study, taking into account sources of potential bias or imprecision. Discuss both direction and magnitude of any potential bias	16, 17
Interpretation	20	Give a cautious overall interpretation of results considering objectives, limitations, multiplicity of analyses, results from similar studies, and other relevant evidence	18
Generalisability	21	Discuss the generalisability (external validity) of the study results	16, 17
**Other information**			
Funding	22	Give the source of funding and the role of the funders for the present study and, if applicable, for the original study on which the present article is based	19

*Give information separately for exposed and unexposed groups.

Note: An Explanation and Elaboration article discusses each checklist item and gives methodological background and published examples of transparent reporting. The STROBE checklist is best used in conjunction with this article (freely available on the Web sites of PLoS Medicine at http://www.plosmedicine.org/, Annals of Internal Medicine at http://www.annals.org/, and Epidemiology at http://www.epidem.com/). Information on the STROBE Initiative is available at http://www.strobe-statement.org.

For peer review only

BMJ Open

Validation of oxygen saturations measured in the community by emergency medical services as a marker of clinical deterioration in patients with confirmed COVID-19

Journal:	*BMJ Open*
Manuscript ID	bmjopen-2022-067378.R1
Article Type:	Original research
Date Submitted by the Author:	23-Jun-2023
Complete List of Authors:	Inada-Kim, Matthew; Hampshire Hospitals NHS Foundation Trust, Department of Acute Medicine<br>Chmiel, Francis P.; University of Southampton<br>Boniface, Michael; University of Southampton<br>Burns, Daniel; University of Southampton<br>Pocock, Helen ; South Central Ambulance Service NHS Foundation Trust; University of Warwick<br>Black, John; South Central Ambulance Service NHS Foundation Trust; Oxford University Hospitals NHS Foundation Trust, Emergency Department<br>Deakin, Charles ; South Central Ambulance Service NHS Foundation Trust; University Hospital Southampton NHS Foundation Trust
**Primary Subject Heading**:	Emergency medicine
Secondary Subject Heading:	Epidemiology, Respiratory medicine
Keywords:	COVID-19, RESPIRATORY MEDICINE (see Thoracic Medicine), Epidemiology < INFECTIOUS DISEASES

SCHOLARONE™
Manuscripts

I, the Submitting Author has the right to grant and does grant on behalf of all authors of the Work (as defined in the below author licence), an exclusive licence and/or a non-exclusive licence for contributions from authors who are: i) UK Crown employees; ii) where BMJ has agreed a CC-BY licence shall apply, and/or iii) in accordance with the terms applicable for US Federal Government officers or employees acting as part of their official duties; on a worldwide, perpetual, irrevocable, royalty-free basis to BMJ Publishing Group Ltd ("BMJ") its licensees and where the relevant Journal is co-owned by BMJ to the co-owners of the Journal, to publish the Work in this journal and any other BMJ products and to exploit all rights, as set out in our licence.

The Submitting Author accepts and understands that any supply made under these terms is made by BMJ to the Submitting Author unless you are acting as an employee on behalf of your employer or a postgraduate student of an affiliated institution which is paying any applicable article publishing charge ("APC") for Open Access articles. Where the Submitting Author wishes to make the Work available on an Open Access basis (and intends to pay the relevant APC), the terms of reuse of such Open Access shall be governed by a Creative Commons licence – details of these licences and which Creative Commons licence will apply to this Work are set out in our licence referred to above.

Other than as permitted in any relevant BMJ Author's Self Archiving Policies, I confirm this Work has not been accepted for publication elsewhere, is not being considered for publication elsewhere and does not duplicate material already published. I confirm all authors consent to publication of this Work and authorise the granting of this licence.

For peer review only

Validation of oxygen saturations measured in the community by emergency medical services as a marker of clinical deterioration in patients with confirmed COVID-19

Matthew Inada-Kim[1]

Francis P. Chmiel[2]

Michael J. Boniface[2]

Daniel K. Burns[2]*

Helen Pocock[3,4]

John J. M. Black[3,5]

Charles D. Deakin[3,6]

[1] Acute Medical Unit, Department of Acute Medicine, Hampshire Hospitals NHS Foundation Trust, Winchester, United Kingdom.
[2] School of Electronics and Computer Science, University of Southampton, Southampton, SO17 1BJ, UK
[3] South Central Ambulance Service NHS Foundation Trust, Otterbourne, SO21 2RU, UK
[4] Warwick Clinical Trials Unit, University of Warwick, Coventry, CV4 7AL, UK
[5] Emergency Department, Oxford University Hospitals NHS Foundation Trust OX3 9DU.
[6] Southampton Respiratory Biomedical Research Unit, National Institute for Health Research, University Hospital Southampton, SO16 6YD

* Corresponding author: d.burns@soton.ac.uk

For peer review only

ABSTRACT

Objectives To evaluate oxygen saturation and vital signs measured in the community by emergency medical services (EMS) as clinical markers of COVID-19-positive patient deterioration.

Design A retrospective data analysis.

Setting Patients conveyed by EMS to two hospitals in Hampshire, UK between March 1st and July 31st 2020.

Participants A total of 1,080 patients aged >= 18 years old with a COVID-19 diagnosis who were conveyed by EMS to hospital.

Primary and secondary outcome measures The primary study outcome was admission to ICU within 30-days of conveyance with a secondary outcome representing mortality within 30-days of conveyance. ROC analysis was performed to evaluate, in a retrospective fashion, the efficacy of different variables in predicting patient outcomes.

Results Vital signs measured by EMS staff at first point of contact in the community correlated with patient 30-day ICU admission and mortality. Oxygen saturation was comparably predictive of 30-day ICU admission (AUROC 0.753 (95 % CI: 0.668-0.826)) to the NEWS2 score (AUROC 0.731 (95 % CI: 0.655-0.800), followed by temperature (AUROC 0.720 (95 % CI: 0.640-0.793)), and respiration rate (AUROC 0.672 (95 % CI: 0.586-0.756)).

Conclusions Initial oxygen saturation measurements (on air) for confirmed COVID-19 patients conveyed by EMS correlated with short-term patient outcomes, demonstrating an AUROC of 0.753 (95% CI: 0.668-0.826) in predicting 30-day ICU admission. We found that threshold of 93% Sp02 is prognostic of adverse events and of value for clinician decision making with sensitivity (74.2 % CI 0.642-0.840) and specificity (70.6 % CI 0.678-0.734).

==

ARTICLE SUMMARY

Strengths and limitations

- This is the first study to report that baseline (community) oxygen saturation measurements (on air) for confirmed COVID-19 patients conveyed by EMS to hospital for further management correlated with short-term (30-day) ICU admission and/or mortality.
- This study assessed vital signs and demographics as predictive factors for short-term (30-day) ICU admission and/or mortality.
- The study has a number of limitations due to data availability, as such we did not include data from patients who were reviewed by EMS but not conveyed, and do not consider the type of measurement device in our analysis

For peer review only

For peer review only - http://bmjopen.bmj.com/site/about/guidelines.xhtml

INTRODUCTION

Severe acute respiratory syndrome coronavirus 2 (SARS-CoV-2) is a highly transmissible and pathogenic coronavirus that causes coronavirus disease 2019 (COVID-19) [1]. COVID-19 presents the biggest global healthcare challenge of our generation. As of February 2021, COVID-19 associated mortality stands at over 110,000 in the UK [2] COVID-19 presents a number of challenges in identifying optimal management pathways, not only in terms of the clinical care itself, but also identifying the stage at which hospital admission is necessary. Traditional management pathways involving paramedic assessment and conveyance to the Emergency Department (ED) for further review have proven impractical, not only because of the large numbers of patients involved, but because of the need to minimise contact of COVID-19 patients with others. Most patients who become symptomatic do so in a home environment where the majority will remain. In terms of optimising outcome, there is a need to understand which symptoms and signs in this environment are prognostic indicators of potential deterioration. The national recommendation for the implementation of COVID virtual wards recently announced by NHS England,[3] ushers in a novel approach of empowering patients through providing symptomatic, at risk patients a pulse oximeter and a toolkit for self-monitoring at home. It is hoped that this will enable the earlier recognition of deterioration in COVID-19 patients and potentially improved outcomes.

In most cases of bacterial and non-COVID pneumonia, breathlessness appears relatively early in the disease and ahead of any significant hypoxia. The challenge with assessing COVID-19 severity is that asymptomatic hypoxia often precedes breathlessness and by the time symptoms of breathlessness occur, patients have developed advanced disease and hypoxia may be significant.[4] The ability to detect this asymptomatic hypoxia before patients experience shortness of breath is critical for preventing respiratory involvement progressing to a life-threatening state. The key is to be able to detect this initial drop in oxygen saturation levels so that patients infected with COVID-19 who begin to suffer from pulmonary complications in the community can be detected early and conveyed to hospital for further treatment.[5] Although some studies have reported the relationship between oxygen saturation and outcome on presentation to the ED, we are not aware of any studies that have reported the relationship between oxygen saturations measured in the community by EMS and outcome. Patients who on assessment are severely hypoxic are clearly in need of emergency conveyance and hospital treatment, but by far the majority of patients with Covid-like symptoms seen and assessed by the EMS have relatively normal or near-normal oxygen saturations. These patients have generally not been conveyed and have been managed at home, but it has become apparent that even relatively minor derangements in oxygen saturations may be an early warning

For peer review only

indicator for disease progression and the subsequent need for critical care. Use of oxygen saturation as an indicator of disease severity may therefore underestimate the risk of leaving patients at home after assessment by the EMS. National case fatality rates (CFR) (ratio of deaths to total cases) have shown a strong inverse correlation between target oxygen saturation levels of 90-98% [6] suggesting that even mild derangements in oxygen saturation untreated can be detrimental to outcome.

Two small studies have suggested the utility of home oxygen monitoring for COVID-19 patients discharged from hospital,[7, 8] but no studies to our knowledge have used out-of-hospital oxygen saturation measurements as a trigger for initial hospital assessment. The purpose of this study therefore is to understand the prognostic significance of oxygen saturation when first measured by EMS clinicians. The understanding aims to inform escalation policies for safe and effective community-based triage and self-monitoring at home by identify a threshold where the sensitivity and specificity are of clinical value. It is hoped that the approach will contribute to hospital admission avoidance, enable earlier recognition of deterioration in COVID-19 patients and potentially improve outcome through early identification of those most at risk of disease progression. Whilst using a pulse oximeter provides a way for patients to monitor disease progression through a simple measurement procedure in contrast to the complexity of measurements required to calculate a NEWS2 score.

For peer review only - http://bmjopen.bmj.com/site/about/guidelines.xhtml

METHODS

Study Design

We undertook a retrospective review of clinically confirmed COVID-19 patients accessing a regional UK ambulance service who were conveyed to hospital and correlated their initial oxygen saturations measured at home with their in-hospital outcome. These were compared with the standard NEWS2 patient score, as used by all UK ambulance services, to identify the deteriorating patient.[9]

The cohort included adult patients (aged 18 years of older) initially assessed and conveyed by personnel from South Central Ambulance Service (SCAS) to the Emergency Department at one of the two hospitals within north Hampshire; Basingstoke & North Hampshire Hospital, or the Royal Hampshire County Hospital (Winchester) at which the patients were subsequently admitted.

The standard care pathway included 1) Patients calling emergency (999) and urgent (111) where they are triaged using NHS Pathways telephone script (release 19), 2) Attendance, assessment and monitoring by ambulance staff at the patient's home, 3) Conveyance to hospital for patients considered at high risk of deterioration 4) Admission to hospital and escalation to ICU for patients requiring critical care.

We analysed EMS conveyances occurring between 1st March to 31st July 2020, to determine suspect COVID-19 among conveyances at initial time of contact by the call taker or EMS staff, each patient record was reviewed for inclusion of at least one of the following four identifiers:

1. Those in who the EMS call taker had classified the call as 'COVID– Respiratory Distress'
2. Those where the Patient Clinical Record (PCR) listed the 'Presenting complaint' as 'Suspected COVID-19'.
3. Those where the PCR free text for the 'Presenting complaint' contained the word 'COVID'
4. Those where the PCR narrative in the free text field summarising the symptoms and their details completed by the paramedic contained the word 'COVID'.

Conveyances from these suspect COVID-19 patients were then linked to their subsequent hospital attendance. Of suspect cases, we then identified confirmed COVID-19 cases by selecting only those with a confirmed diagnosis in their discharge summary (i.e., the presence of a U07.1 or U07.2 ICD10 code). These confirmed COVID-19 cases made up our study cohort.

Seventeen patients did not have initial oxygen saturations recorded on air (but did have oxygen saturations recorded on oxygen) and were excluded from the data analysis. If this was because they were so obviously hypoxic clinically that EMS staff immediately administered oxygen without an

For peer review only - http://bmjopen.bmj.com/site/about/guidelines.xhtml

initial reading on air (or were constantly on home oxygen treatment), the ability of oxygen saturations to indicate risk of deterioration is likely to have been underestimated in this study.

All patients in known palliative care pathways were excluded from data analysis because their care did not follow standard care pathways.

Study setting

SCAS is a provider of emergency care in the counties of Hampshire, Berkshire, Buckinghamshire and Oxfordshire and covers a total of 3554 sq. miles (9205 km^2). The service receives approximately 500,000 emergency and urgent calls annually. SCAS covers a residential population of approximately 4.0 million inhabitants in a mix of urban and rural areas. The north Hampshire region forms part of the area covered by SCAS and comprises a residential population of approximately 306,000.[10]

Data collection

The initial oxygen saturation reading (SpO_2) on air recorded by the attending EMS staff (prior to any exercise or step test) and the NEWS2 score of patients fulfilling the inclusion criteria were collected from the EMS PCR. (NEWS2 score is calculated using the following seven variables: systolic blood pressure, heart rate, respiratory rate, temperature, oxygen saturation, supplemental oxygen administration, and level of consciousness - https://www.england.nhs.uk/ourwork/clinical-policy/sepsis/nationalearlywarningscore.)

Patient outcome was obtained by linking the SCAS and hospital clinical records by their NHS number. The primary outcome of our study was ICU admission within 30-days of conveyance and the secondary outcomes was mortality and a combined outcome (ICU admission and/or mortality) within 30-days of conveyance.

Data analysis

Analysis was performed in Python 3.7.2 [10], primarily making use of the statsmodels library [11]. Confidence intervals on observed mortality rates were estimated using the Wilson score interval. Where relevant, significance of the difference between two observed adverse outcome rates were tested using a two-population proportions z-test with the null hypothesis that the two-population proportions are equal.

For peer review only

For peer review only - http://bmjopen.bmj.com/site/about/guidelines.xhtml

To evaluate how predictive individual variables (e.g., oxygen saturation) and combinations of variables (e.g., oxygen saturation with age) were of 30-day adverse outcomes, we performed Receiving Operator Characteristics curve analysis. In the univariate analysis, we performed a complete case analysis (removing any patient with an incomplete record of vital signs) and assume a patient's adverse outcome risk is a linear function of the respective variable (where negative or positive correlation with outcome is assessed by clinical judgement) and calculated the ROC curve corresponding to if this variable alone was used to predict a patient's risk of an adverse outcome. We present both the sensitivity and specificity or the Area Under the Receiving Operator Characteristic curve (AUROC). The AUROC provides an estimate of the degree to which the predictor can discern between whether a patient has an adverse outcome within 30 days of conveyance or not, it can take values between 0.5 and 1.0. An AUROC of 0.5 corresponds to randomly guessing which patient have an adverse outcome within 30 days and an AUROC of 1.0 corresponds to a perfect classifier - it can predict, without error, who will have an adverse outcome within 30-days of conveyance. Confidence intervals were estimated by performing 1000 bootstrapping (sampling with replacement) iterations on the available data, calculating the AUROC on each of the samples and then calculating the relevant percentiles.

Patient and Public Involvement

This research was done without patient involvement. Patients were not invited to comment on the study design and were not consulted to develop patient-relevant outcomes or interpret the results. Patients were not invited to contribute to the writing or editing of this document for readability or accuracy.

Governance and ethics approval

Regulatory and ethical approval for the study were provided by the Health Research Authority (REC reference 20/HRA/5445) and by the University of Southampton Ethics Committee (REF ERGO/61242). NHS England and NHS Improvement have been given legal notice by the Secretary of State for Health and Social Care to support the processing and sharing of information to help the COVID-19 response under Health Service Control of Patient Information Regulations 2002 (COPI). This is to ensure that confidential patient information can be used and shared appropriately and lawfully for purposes related to the COVID-19 response. Data were extracted from medical records by clinicians providing care for the patients and an anonymised extract of the data were provided to the team at the University of Southampton.

For peer review only

For peer review only - http://bmjopen.bmj.com/site/about/guidelines.xhtml

For peer review only

For peer review only - http://bmjopen.bmj.com/site/about/guidelines.xhtml

RESULTS

A total of 19,868 patients were assessed at home and subsequently conveyed by EMS to North Hampshire Hospitals during the study period. The details of cohort selection are shown in Figure 1. The call handler or EMS staff identified 2,257 suspect COVID-19 cases and of these we identified 1,209 adults as having a confirmed diagnosis of COVID-19 (U07.1 or U07.2 coded in the patients discharge summary). Of the 1,209 confirmed cases we removed persons under palliative care (112 patients) and those with no initial oxygen saturation measurement on air recorded (17 patients). Overall, this left us with 1,080 confirmed COVID-19 patient records all of whom had initial oxygen saturation measurements on air. Of these 1,080, the complete records of vital signs were recorded at home by paramedics for 892 of the patients. The summary of the final patient cohort, with respect to demographics, comorbidities, and presence of vital sign measurements is given in Table 1. In our following discussions, we make use of all 1,080 patients, with the exception for our univariate analyses where we perform a complete case analysis and only use the 892 complete records.

Oxygen saturation was found to correlate with adverse outcome (Figure 2A), with lower initial oxygen saturation readings being associated with a higher mortality rate. In Figure 2A we display the correlation between the observed 30-day adverse outcome rates and initial oxygen saturation in detail, which displays correlation to all outcomes. In Table 2 we display the breakdown of our retrospective ROC analysis for using measured oxygen saturation as a binary triage tool (i.e., hospitalize or not) for different cut-offs (rows in Table 2). While the sensitivity vs specificity trade-off needs to be determined by the clinical context, this demonstrates that oxygen saturation is moderately discriminative for several cut-offs. For example, for a cut-off of 94 % or below, the sensitivity is 0.742 (95 % CI: 0.642-0.734) and the specificity is 0.706 (95 % CI: 0.678-0.734). Finally, we present comparisons of the results of ROC analysis for different variables measured in the community by EMS (Table 3). Across the three presented outcomes (30-day ICU admission, mortality, and combined outcome) correlations between variables and outcomes are broadly similar, with measured oxygen saturations and the NEWS2 score being the two most predictive of outcome. The notable differences are for the measured temperature which is moderately predictive of ICU admission (AUROC: 0.720 (95 % CI: 0.640-0.793)) but only weakly predictive of mortality (AUROC: 0.597 (95 % CI: 0.523-0.678)) and for patient age which is strongly positively correlated to mortality but displays a negative correlation to ICU admission (Figure 2B).

For peer review only

For peer review only - http://bmjopen.bmj.com/site/about/guidelines.xhtml

Variable	Outcome Category		
Outcome	No adverse event(n=955)	30-day ICU admission (n=58)	30-day mortality (n=78)
**Age**			
18-49	159 (16.6%)	11 (19.0%)	1 (1.3%)
50-59	132 (13.8%)	16 (27.6%)	2 (2.6%)
60-69	119 (12.5%)	17 (29.3%)	9 (11.5%)
70-79	209 (21.9%)	9 (15.5%)	16 (20.5%)
80+	336 (35.2%)	5 (8.6%)	50 (64.1%)
**Comorbidities**			
Chronic Obstructive Pulmonary Disorder	33 (3.5%)	0 (0.0%)	6 (7.7%)
Dementia	90 (9.4%)	1 (1.7%)	18 (23.1%)
Diabetes	216 (22.6%)	14 (24.1%)	14 (17.9%)
Kidney disease	7 (0.7%)	1 (1.7%)	3 (3.8%)
Chronic pain	37 (3.9%)	3 (5.2%)	1 (1.3%)
**Vital signs**			
Heart rate present	946 (99.1%)	58 (100.0%)	77 (98.7%)
Systolic blood pressure present	869 (91.0%)	51 (87.9%)	71 (91.0%)
Respiratory rate present	852 (89.2%)	49 (84.5%)	70 (89.7%)
Oxygen saturation (on air) present	955 (100.0%)	58 (100.0%)	78 (100.0%)
Temperature present	825 (86.4%)	49 (84.5%)	67 (85.9%)
ACVPU present	849 (88.9%)	50 (86.2%)	67 (85.9%)

Table 1: Characteristics of COVID-19 positive patients stratified by outcome. Note that n=11 patients experienced both ICU admission and mortality within 30 days. We only report on comorbidities which were present in the dataset as provided by the EMS. Comorbidity presence was recorded for every patient in the study. ACVPU = alert, confused, responding to voice, responding to pain, unresponsive. Oxygen saturations were not missing for any patients as those with missing values had been excluded (n=17). Overall, vital signs records were complete for 83% of cases.

For peer review only - http://bmjopen.bmj.com/site/about/guidelines.xhtml

Oxygen Saturation (on air) threshold (%)	Sensitivity (95 % CI)	Specificity (95 % CI)	Number of observations	Cumulative sum of number of observations
**85**	0.294 (0.200-0.400)	0.947 (0.933-0.962)	8	76
**86**	0.316 (0.216-0.421)	0.941 (0.927-0.955)	8	84
**87**	0.320 (0.216-0.432)	0.935 (0.920-0.950)	6	90
**88**	0.370 (0.261-0.476)	0.916 (0.899-0.933)	23	113
**89**	0.413 (0.304-0.523)	0.894 (0.874-0.913)	25	138
**90**	0.512 (0.411-0.615)	0.870 (0.849-0.890)	32	170
**91**	0.590 (0.477-0.699)	0.841 (0.823-0.867)	31	201
**92**	0.655 (0.544-0.761)	0.817 (0.796-0.841)	33	234
**93**	0.706 (0.593-0.803)	0.776 (0.751-0.801)	45	279
**94**	0.742 (0.642-0.840)	0.706 (0.678-0.734)	74	353
**95**	0.808 (0.718-0.892)	0.634 (0.605-0.662)	76	429
**96**	0.848 (0.767-0.921)	0.508 (0.477-0.538)	129	558
**97**	0.898 (0.822-0.963)	0.357 (0.330-0.386)	156	714
**98**	0.911 (0.841-0.973)	0.226 (0.201-0.254)	132	846
**99**	0.961 (0.913-1.0)	0.091 (0.075-0.109)	139	985
**100**	1	0	95	1080

Table 2: Evaluation of initial oxygen saturation measured by paramedics in COVID-19 patients in the community used as a binary classifier for predicting 30-day ICU admission within 30 days of conveyance. Each row denotes a different threshold for determining those at risk of an adverse outcome. We display the sensitivity and specificity for each threshold, equivalent to all possible intersections of the receiving operator curve using thresholds between 85 % and 100 %. In total 68 patients had an oxygen saturation of 84 % or less (not shown). The column on the far right denotes the cumulative sum of the number of observations of the given oxygen saturation (row) or below. For example, 76 patients had an oxygen saturation of 85 % or less recorded (top row) and 429 patients had an oxygen saturation of 95 % or less recorded. Confidence intervals are estimated by bootstrapping.

For peer review only

For peer review only - http://bmjopen.bmj.com/site/about/guidelines.xhtml

	AUROC (95 % CI)		
Variable	ICU admission	Mortality	Combined
**Oxygen Saturation (on air)**	0.753 (0.668-0.826)	0.778 (0.704-0.843)	0.775 (0.727-0.829)
**NEWS2**	0.731 (0.655-0.800)	0.768 (0.709-0.823)	0.760 (0.708-0.807)
**Respiration rate**	0.672 (0.586-0.756)	0.668 (0.599-0.736)	0.677 (0.618-0.738)
**Temperature**	0.720 (0.640-0.793)	0.597 (0.523-0.678)	0.636 (0.69-0.700)
**Systolic blood pressure**	0.634 (0.560-0.706)	0.604 (0.529-0.680)	0.626 (0.568-0.684)
**Heart rate**	0.590 (0.506-0.672)	0.558 (0.486-0.631)	0.574 (0.514-0.633)
**Age band**	0.670 (0.611-0.734)	0.685 (0.626-0.738)	0.557 (0.495-0.615)

Table 3: Ranked Area Under Receiver Operator Curves (AUROC) calculated for isolated physiological variables and the composite NEWS2 score with each outcome. AUROCS were calculated using a complete case analysis with 892 patients in total. Confidence intervals are estimated by bootstrapping, with 95 % confidence intervals presented alongside the mean validation AUROC across samples.

For peer review only

For peer review only - http://bmjopen.bmj.com/site/about/guidelines.xhtml

For peer review only

DISCUSSION

Community assessment of patients with COVID-19 symptoms using a single initial oxygen saturation on air measurement correlates with 30-day clinical outcomes. Qualitatively, the observed 30-day adverse outcome rate is approximately constant between oxygen saturations of 100 - 96 % and then increase with decreasing oxygen saturation from 95 % to 90 %. Below 90 %, the mortality risk remains high. Although the therapeutic target range for oxygen saturations in the UK is 94-98%,[11] and in the USA is 92-96%,[12] this study suggests that patients at the lower end of this range are still at risk of deterioration in the context of COVID-like symptoms. For example, for patients in our cohort with presenting oxygen saturations in the range of 92-94 %, values often regarded as within this normal range, had a significantly (p=0.025) higher risk of ICU admission within 30 days (5.9 %) compared to those presenting with oxygen saturations greater than 95 % (ICU admission rate 2.5 %). Outside this 'normal' range, our analysis suggests even relatively small decreases in oxygen saturation are markers of increased risk of death or ICU admission and suggest that a lower threshold for hospital conveyance may be necessary for patients who traditionally would be considered to have only minor physiological derangement and otherwise have been left at home.

The sensitivity of home oxygen saturation measurements reflects the percentage of people correctly identified with adverse outcomes. The sensitivity of this parameter for adverse outcome decreased as oxygen saturation fell (Table 2). An oxygen saturation ≤ 90% was associated with a relatively low sensitivity of < 0.5. Specificity of identifying an adverse outcome, an indirect measure of unnecessary conveyance to hospital (but also including patients who survived and did not need ICU admissions), increased as oxygen saturations fell. However, it is important to ensure that patients at risk of deterioration are not missed and a degree of over-triage would be necessary to ensure that this was not the case. However, even oxygen saturations at the lower end of the normal range are associated with a risk of deterioration (sensitivity of 94% saturations = 0.713) and it therefore appears that oxygen saturation alone has significant limitations when it is within a normal range.

Although oxygen saturations as a risk factor for COVID-19 patients on presentation to the Emergency Department are widely reported,[13, 14] the ability of oxygen saturations measured in the community to indicate disease severity and the need for hospital conveyance has not been widely reported, presumably because of the challenges in equipping patients with pulse oximeters prior to the onset of any illness. Several studies have used oxygen levels in patients presenting in the ED as an indicator of the need for hospital admission and others have used the opportunity to send ED patients not requiring admission home with a pulse oximeter for self-monitoring. Oxygen saturations on presentation to the ED have also been shown to be strongly associated with outcome. The

For peer review only

For peer review only - http://bmjopen.bmj.com/site/about/guidelines.xhtml

strongest critical illness risk has been shown to be admission oxygen saturation < 88% (OR 6.99).[14] Other studies have shown that even a relatively mildly deranged oxygen saturation of <92% is strongly associated with an increased risk of in-hospital mortality.[15] Conversely, an ED resting SpO_2 ≥ 92% as part of discharge criteria can achieve hospital readmission rates as low as 4.6%, [16] suggesting that it may be a safe threshold for discharge in symptomatic patients with mild disease after diagnostic workup .

Home oxygen saturation monitoring has been used for patients discharged from hospital, either from the ED because their disease was not severe, or from intensive care for convalescence. A small study of patients with COVID-19 discharged from an ED, reported similar results to ours using subsequent home oxygen saturation monitoring. In these patients, resting home SpO_2 < 92% was associated with an increased likelihood of re-hospitalization compared to SpO_2 ≥ 92% (relative risk = 7.0, 95% CI 3.4 to 14.5, p < 0.0001). Home SpO_2 < 92% was also associated with increased risk of intensive care unit admission.[8]

Oxygen saturation is an integral variable in most critical illness tools. The association of prehospital oxygen saturation has been shown to be predictive of 2-day mortality [17]and has been used to identify COVID-19 patients requiring hospital admission.[18] NHS England has encouraged the use of the NEWS2 scoring system to identify patients at risk of deterioration. This uses weighted physiological variables of heart rate, systolic blood pressure, oxygen saturation (on air), respiratory rate, temperature and level of consciousness to produce a score that is correlated with risk of deterioration, not only as a general illness score, but specifically in patients with known COVID-19.[19] NEWS2 has been compared with a quick COVID Sensitivity Index (qCSI), a test that includes SpO2, respiratory rate and O2 flow rate to calculate a score between 1 and 12, and risk level. The study concludes NEWS2 is significantly better than qCSI, with AUC of 0.779 and 0.750 respectively [20]. Furthermore, qCSI does not consider severity score for readings of 93% and above, whilst qCSI pulse oximetry readings are the lowest reading recorded during the first 4 hours of patient encounter at the hospital, rather than being prior to admission. In our study, we were concerned with the ability of isolated oxygen saturations measured by EMS on attendance in comparison with NEWS2 in our cohort to identify patients at risk of ICU admission (and mortality) within 30 days. Using ROC analysis, the AUROC for oxygen saturations at predicting ICU admission alone was 0.753 (95% CI 0.668-0.826) and for NEWS2 was 0.731 (95% CI 0.655-0.800). These results are consistent with a previous study using NEWS2 scores on hospital admission which has shown an AUROC of 0.822 (95% CI 0.690-0.953) to predict risk of severe disease.[19] The lower observed AUROC of NEWS2 compared to oxygen saturations may be the result of the NEWS2 score incorporating

For peer review only

physiological variables less predictive of COVID-19 outcomes than oxygen saturations, thereby reducing the discriminative ability of the score, or because it uses discretized oxygen saturations which amounts to information loss. Additionally, we have not assessed the reporting compliance of the NEWS2 scores and this may have impacted the observed AUROCs. Interestingly, a recent review of 22 prognostic models showed that oxygen saturation on room air and patient age were strong predictors of deterioration and mortality among hospitalised adults with COVID-19 respectively, but no other variables added incremental value to these predictors.[18] We have shown the same for oxygen saturation as a univariate predictor in the pre-hospital setting, and that predictive value does not increase by the addition of other physiological variables. The PRIEST study using NEWS2, age, sex, and performance status of patients in the ED predicted adverse outcome with good discrimination in adults with suspected COVID-19 [20]. The discriminatory ability of this more complex scoring system was similar to that demonstrated by simply measuring the oxygen saturations in the community and further reinforces the utility of home oxygen saturations as a simple marker, not only for use by the EMS, but by members of the public equipped with home oximetry.

A number of remote home monitoring models for patients with suspected COVID-19 have been proposed, all of which aim to achieve early identification of deterioration for patients self-managing COVID-19 symptoms at home.[21] It would be expected that the utility of home monitoring would be improved by the ability to measure oxygen saturations, although not all models currently integrate this into their protocols. Our results show that resting oxygen saturations measured in patients with confirmed COVID-19 perform on a par with the same measurements taken in the ED. They therefore suggest that the predictive value of oxygen saturations may be able to be effectively moved to an earlier stage in the disease process and measured while the patient is still at home. Although initial home SpO_2 may provide a useful marker of disease severity and the need for hospital conveyance, it is clear that it has limited sensitivity and may need to be interpreted as part of an overall assessment of the patient. Some authors have argued that pulse oximetry identified the need for hospitalisation when using a cut- off of 92%,[8] but based on our data (Table 2), approximately one-third of patients with an adverse outcome would be missed using this threshold. We have demonstrated that even patients presenting with oxygen saturations of 92-94 %, which are values often regarded as within a normal range, have a higher mortality than those with oxygen saturations higher than 95 %. Even when measured in the ED, baseline median SpO_2 was as high as 95.0 % in those with an adverse outcome, compared to 97.0% in those without.[22] It is clear that

For peer review only - http://bmjopen.bmj.com/site/about/guidelines.xhtml

the relatively low sensitivity of oxygen saturation in those with mildly deranged values limits the utility of this parameter alone in assessing risk of adverse outcome.

This is a relatively small retrospective cohort study with concomitant limitations of sample size. The subjective nature of paramedic classification of symptoms consistent with COVID-19 may have introduced some degree of bias into patients included in the study, as may have the presence of known co-morbidities. Our dataset did not include patients who were reviewed by EMS but not conveyed to hospital and this is arguably the most significant source of bias in our study. It is reasonable that for patients where a decision was made not to convey them, they were less likely to deteriorate and more likely to have normal vital signs. If this is the case, this would result in a reduction of the discriminative ability of recorded oxygen saturations. We did not specifically compare the outcome data of COVID and Non-COVID patients with mildly deranged oxygen saturations. However, our data suggests that mild derangement in COVID patients is a significant risk factor for deterioration and this does not match the clinical progression witnessed in non-COVID patients. We acknowledge that for very low Sp02 levels our results show poor clinical value and we believe this is due to other factors influencing escalation decisions that are not included in our dataset.   Patients on palliative care pathways were also removed from the study cohort, but are likely to be more susceptible to deterioration from COVID, irrespective of any alternative care pathway.

With waves of COVID-19 regularly overwhelming EMS and hospital services, there is an urgent need to optimise the identification of patients at risk of deterioration. We undertook this research to ascertain the role simple physiological measures might have to inform clinical decision making. While the results are hypothesis-forming (i.e., it shows oxygen saturations are predictive of clinical outcomes within the care pathway studied in this manuscript), it has clinical utility as it helps inform decisions made by clinicians at the point of conveyance. This will enable more patients to be safely managed in the community and only referred to hospital once their clinical symptoms and physiological signs suggest a risk of deterioration and the need for hospital care. This is particularly needed for the majority of patients who have mild to moderate symptoms where it is not clear if community or hospital management is appropriate. Home pulse oximetry is becoming relatively cheap and easily accessible for the public and may be a relatively cost-effective tool in the safe community management of these patients, perhaps focussed on those with significant co-morbidities who are at higher risk. The utility of remote monitoring systems (or the COVID virtual ward) has been an increasingly studied subject, and there is growing evidence that remote

For peer review only - http://bmjopen.bmj.com/site/about/guidelines.xhtml

monitoring can facilitate more streamlined approaches to the delivery of patient care, especially in pulmonary disease.[7] The use of ICU admission as an endpoint identifies patients seen at home who go on to deteriorate and the correlation of home oxygen saturation with a risk of severe deterioration assists ambulance crews in identifying both those who should be conveyed to hospital as well as those who can, with a reasonable degree of certainty, be safely left at home. Further prospective studies are required to understand the utility of home pulse oximetry, but this study suggests that it may have the potential to significantly contribute to the safe and appropriate management of these patients in the community with timely referral to hospital when indicated.

Conclusions

We have demonstrated that even relatively minor derangements in peripheral oxygen saturation are an early warning of potential deterioration in confirmed COVID-19 patients conveyed by EMS to hospital and oxygen saturation would appear to have potential to be a key physiological variable that together with other clinical signs and clinical risk factors may be able to identify patients at risk of deterioration.

Acknowledgements

We thank Simon Mortimore and Philip King from South Central Ambulance Service and Zoe Cameron from Hampshire Hospitals NHS Foundation Trust for their assistance in data extraction and analysis.

Funding statement

This report includes independent research funded by the National Institute for Health Research Applied Research Collaboration Wessex. The views expressed in this publication are those of the author(s) and not necessarily those of the National Institute for Health Research or the Department of Health and Social Care.

Author contributions

MI-K, MJB, JJM Black, CDD led and conceptualized the study. MI-K led at HHFT, MJB led at UoS, CDD led at SCAS. FPC and DKB performed the data analysis with support and guidance from all authors. MI-K, HP and JJM Black performed the data extraction. MJB led the data governance. CDD and HP provided clinical insight. MI-K, CDD, HP and FPC wrote the first draft of the manuscript. All authors discussed the results. All others contributed to subsequent drafts of the manuscript. DKB prepared the final manuscript for submission.

For peer review only

For peer review only - http://bmjopen.bmj.com/site/about/guidelines.xhtml

Competing interests

M. I-K. is National Clinical Lead Deterioration & National Specialist Advisor Sepsis, NHS England and NHS Improvement. All other authors declare no competing interests.

Data accessibility

Due to information governance concerns, the data will not be made public. However, it will be made accessible via reasonable request to the corresponding author.

For peer review only

For peer review only - http://bmjopen.bmj.com/site/about/guidelines.xhtml

References

1. Hu, B., et al., *Characteristics of SARS-CoV-2 and COVID-19.* Nature Reviews Microbiology, 2021. **19**(3): p. 141-154.
2. England, P.H. *Coronavirus (COVID-19) in the UK*. Available from: https://coronavirus.data.gov.uk.
3. *Pulse oximetry to detect early deterioration of patients with COVID-19 in primary and community care settings, 11 June 2020, Version 1, NHS. https://www.england.nhs.uk/coronavirus/publication/pulse-oximetry-to-detect-early-deterioration-of-patients-with-covid-19-in-primary-and-community-care-settings Accessed 28 Oct 2020.*
4. Bickler, P.E., et al., *"Silent" Presentation of Hypoxemia and Cardiorespiratory Compensation in COVID-19.* Anesthesiology, 2020.
5. Teo, J., *Early Detection of Silent Hypoxia in Covid-19 Pneumonia Using Smartphone Pulse Oximetry.* J Med Syst, 2020. **44**(8): p. 134.
6. Goyal, D., Donnelly H., Kussner, A., Bhatti, N.J., Mansab, F., *Oxygen and mortality in COVID-19 pneumonia: a comparative analysis of supplemental oxygen policies and health outcomes across 26 countries.* 2020.
7. O'Carroll, O., et al., *Remote monitoring of oxygen saturation in individuals with COVID-19 pneumonia.* Eur Respir J, 2020. **56**(2).
8. Shah, S., et al., *Novel use of home pulse oximetry monitoring in COVID-19 patients discharged from the emergency department identifies need for hospitalization.* Acad Emerg Med, 2020.
9. *Royal College of Physicians National Early Warning Score (NEWS) 2. London: RCP, 2017. www.rcplondon.ac.uk/projects/outputs/national-early-warning-score-news-2 [Accessed 19 Oct 2020].*
10. *https://www.hants.gov.uk/landplanningandenvironment/facts-figures/population/estimates-forecasts. Accessed 17th October 2020.*
11. O'Driscoll, B.R., et al., *BTS guideline for oxygen use in adults in healthcare and emergency settings.* Thorax, 2017. **72**(Suppl 1): p. ii1-ii90.
12. *COVID-19 Treatment Guidelines Panel. Coronavirus Disease 2019 (COVID-19) Treatment Guidelines. National Institutes of Health. Available at https://www.covid19treatmentguidelines.nih.gov/. Accessed 15th October 2020.*
13. Gidari, A., et al., *Predictive value of National Early Warning Score 2 (NEWS2) for intensive care unit admission in patients with SARS-CoV-2 infection.* Infect Dis (Lond), 2020. **52**(10): p. 698-704.
14. Petrilli, C.M., et al., *Factors associated with hospital admission and critical illness among 5279 people with coronavirus disease 2019 in New York City: prospective cohort study.* BMJ, 2020. **369**: p. m1966.
15. Mikami, T., et al., *Risk Factors for Mortality in Patients with COVID-19 in New York City.* J Gen Intern Med, 2020.
16. Berdahl, C.T., et al., *The safety of home discharge for low-risk emergency department patients presenting with coronavirus-like symptoms during the COVID-19 pandemic: A retrospective cohort study.* JACEPOPEN, 2020. **August 2020. https://doi.org/10.1002/emp2.12230**.
17. Martín-Rodríguez, F., et al., *Association of Prehospital Oxygen Saturation to Inspired Oxygen Ratio With 1-, 2-, and 7-Day Mortality.* JAMA Network Open, 2021. **4**(4): p. e215700-e215700.
18. Gupta, R.K., et al., *Systematic evaluation and external validation of 22 prognostic models among hospitalised adults with COVID-19: An observational cohort study.* Eur Respir J, 2020.

For peer review only - http://bmjopen.bmj.com/site/about/guidelines.xhtml

19. Myrstad, M., et al., *National Early Warning Score 2 (NEWS2) on admission predicts severe disease and in-hospital mortality from Covid-19 - a prospective cohort study.* Scand J Trauma Resusc Emerg Med, 2020. **28**(1): p. 66.

20. Martín-Rodríguez, F., et al., *One-on-one comparison between qCSI and NEWS scores for mortality risk assessment in patients with COVID-19.* Annals of Medicine, 2022. **54**(1): p. 646-654.

21. Vindrola-Padros, C., et al., *Remote home monitoring (virtual wards) during the COVID-19 pandemic: a living systematic review. 2020.* https://www.medrxiv.org/content/10.1101/2020.10.07.20208587v1.full.pdf.

22. *Goodacre, S. , Thomas, B., Lee, E. et al. Post-exertion oxygen saturation as a prognostic factor for adverse outcome in patients attending the emergency department with suspected COVID-19 : observational cohort study. medRxiv. (Submitted:2020).*

For peer review only

For peer review only

For peer review only - http://bmjopen.bmj.com/site/about/guidelines.xhtml

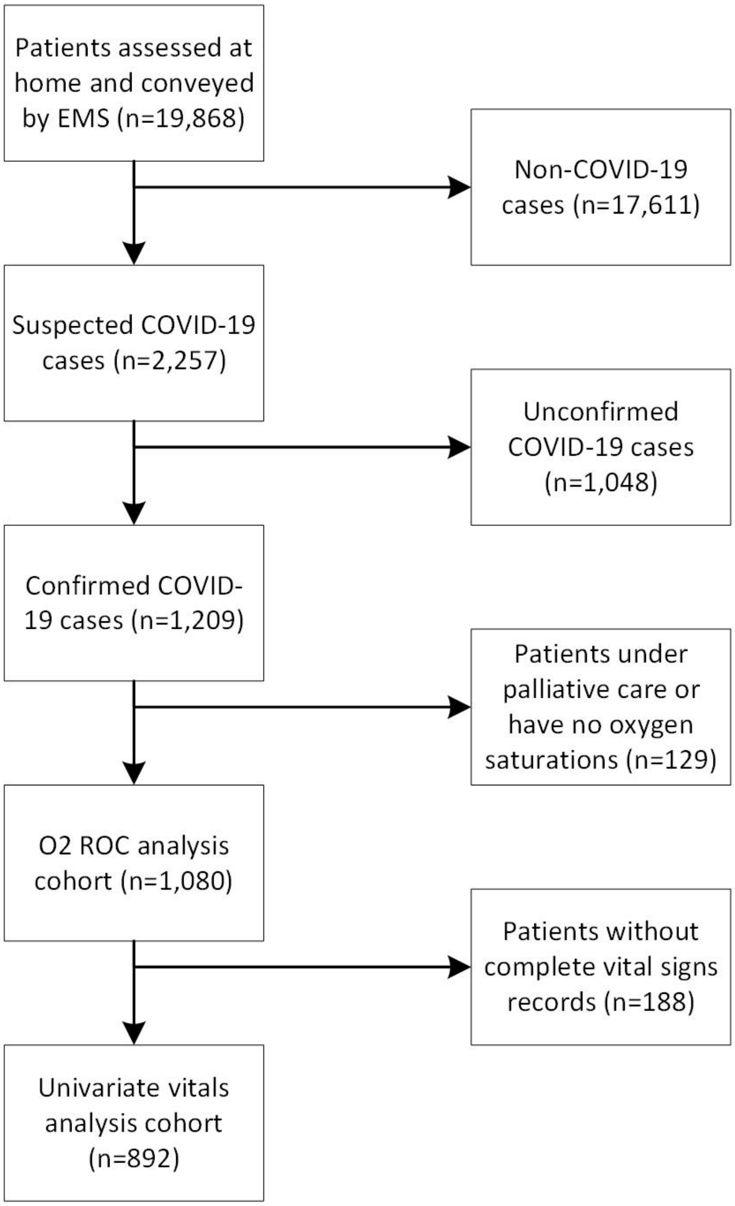

The cohort selection of the EMS patients.

71x115mm (300 x 300 DPI)

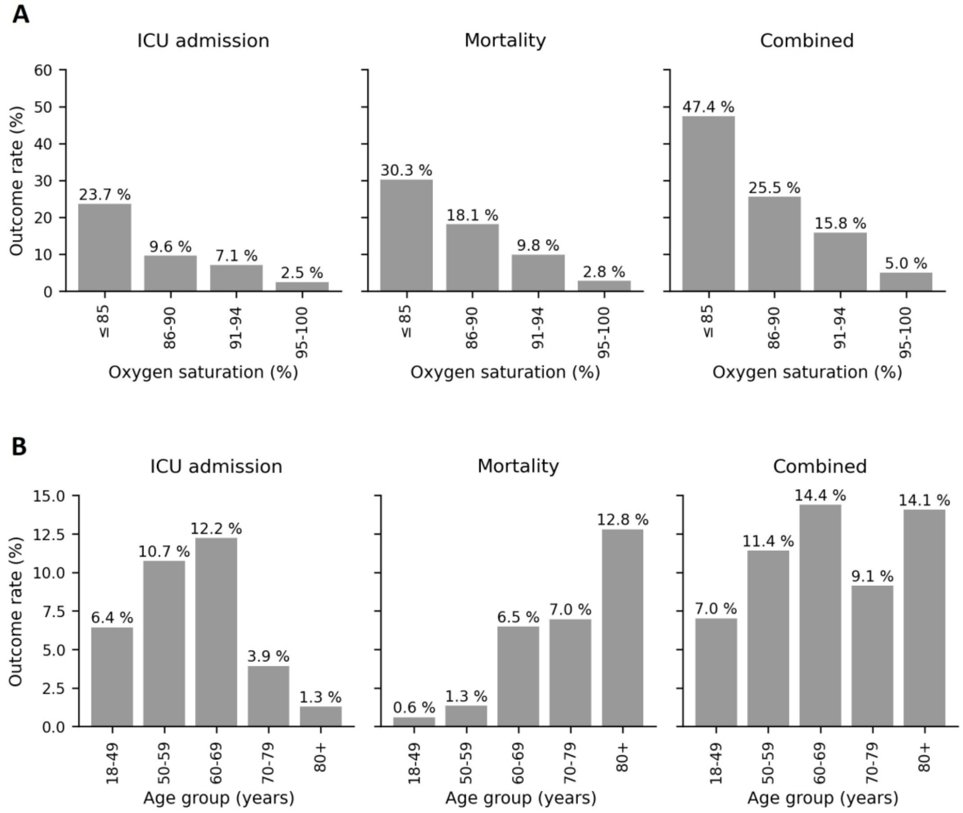

A) The ICU admission, mortality, and combined outcome rates as a function of oxygen saturation %. B) The ICU admission, mortality, and combined outcome rates as a function of age group.

188x166mm (300 x 300 DPI)

STROBE Statement—Checklist of items that should be included in reports of ***cohort studies***

	Item No	Recommendation	Page No
**Title and abstract**	1	(*a*) Indicate the study's design with a commonly used term in the title or the abstract	1
		(*b*) Provide in the abstract an informative and balanced summary of what was done and what was found	2
**Introduction**			
Background/rationale	2	Explain the scientific background and rationale for the investigation being reported	5
Objectives	3	State specific objectives, including any prespecified hypotheses	6
**Methods**			
Study design	4	Present key elements of study design early in the paper	7
Setting	5	Describe the setting, locations, and relevant dates, including periods of recruitment, exposure, follow-up, and data collection	7, 8
Participants	6	(*a*) Give the eligibility criteria, and the sources and methods of selection of participants. Describe methods of follow-up	7, 8
		(*b*) For matched studies, give matching criteria and number of exposed and unexposed	
Variables	7	Clearly define all outcomes, exposures, predictors, potential confounders, and effect modifiers. Give diagnostic criteria, if applicable	8, 9
Data sources/ measurement	8*	For each variable of interest, give sources of data and details of methods of assessment (measurement). Describe comparability of assessment methods if there is more than one group	8, 9
Bias	9	Describe any efforts to address potential sources of bias	16
Study size	10	Explain how the study size was arrived at	N/A
Quantitative variables	11	Explain how quantitative variables were handled in the analyses. If applicable, describe which groupings were chosen and why	8, 9
Statistical methods	12	(*a*) Describe all statistical methods, including those used to control for confounding	8, 9, 10
		(*b*) Describe any methods used to examine subgroups and interactions	
		(*c*) Explain how missing data were addressed	
		(*d*) If applicable, explain how loss to follow-up was addressed	
		(*e*) Describe any sensitivity analyses	
**Results**			
Participants	13*	(a) Report numbers of individuals at each stage of study—eg numbers potentially eligible, examined for eligibility, confirmed eligible, included in the study, completing follow-up, and analysed	7, 10
		(b) Give reasons for non-participation at each stage	
		(c) Consider use of a flow diagram	
Descriptive data	14*	(a) Give characteristics of study participants (eg demographic, clinical, social) and information on exposures and potential confounders	Figures 1, 2, 3 Table 1
		(b) Indicate number of participants with missing data for each variable of interest	Pages 11, 12, 13
		(c) Summarise follow-up time (eg, average and total amount)	

For peer review only - http://bmjopen.bmj.com/site/about/guidelines.xhtml

Outcome data	15*	Report numbers of outcome events or summary measures over time	10
Main results	16	(*a*) Give unadjusted estimates and, if applicable, confounder-adjusted estimates and their precision (eg, 95% confidence interval). Make clear which confounders were adjusted for and why they were included<br>(*b*) Report category boundaries when continuous variables were categorized<br>(*c*) If relevant, consider translating estimates of relative risk into absolute risk for a meaningful time period	10
Other analyses	17	Report other analyses done—eg analyses of subgroups and interactions, and sensitivity analyses	11, 12, 13
**Discussion**			
Key results	18	Summarise key results with reference to study objectives	14, 15
Limitations	19	Discuss limitations of the study, taking into account sources of potential bias or imprecision. Discuss both direction and magnitude of any potential bias	16, 17
Interpretation	20	Give a cautious overall interpretation of results considering objectives, limitations, multiplicity of analyses, results from similar studies, and other relevant evidence	18
Generalisability	21	Discuss the generalisability (external validity) of the study results	16, 17
**Other information**			
Funding	22	Give the source of funding and the role of the funders for the present study and, if applicable, for the original study on which the present article is based	19

*Give information separately for exposed and unexposed groups.

Note: An Explanation and Elaboration article discusses each checklist item and gives methodological background and published examples of transparent reporting. The STROBE checklist is best used in conjunction with this article (freely available on the Web sites of PLoS Medicine at http://www.plosmedicine.org/, Annals of Internal Medicine at http://www.annals.org/, and Epidemiology at http://www.epidem.com/). Information on the STROBE Initiative is available at http://www.strobe-statement.org.

For peer review only

BMJ Open

Validation of oxygen saturations measured in the community by emergency medical services as a marker of clinical deterioration in patients with confirmed COVID-19 - a retrospective cohort study

Journal:	*BMJ Open*
Manuscript ID	bmjopen-2022-067378.R2
Article Type:	Original research
Date Submitted by the Author:	20-Nov-2023

Complete List of Authors:	Inada-Kim, Matthew; Hampshire Hospitals NHS Foundation Trust, Department of Acute Medicine<br>Chmiel, Francis P.; University of Southampton<br>Boniface, Michael; University of Southampton<br>Burns, Daniel; University of Southampton<br>Pocock, Helen ; South Central Ambulance Service NHS Foundation Trust; University of Warwick<br>Black, John; South Central Ambulance Service NHS Foundation Trust; Oxford University Hospitals NHS Foundation Trust, Emergency Department<br>Deakin, Charles ; South Central Ambulance Service NHS Foundation Trust; University Hospital Southampton NHS Foundation Trust

**Primary Subject Heading**:	Emergency medicine
Secondary Subject Heading:	Epidemiology, Respiratory medicine
Keywords:	COVID-19, RESPIRATORY MEDICINE (see Thoracic Medicine), Epidemiology < INFECTIOUS DISEASES

SCHOLARONE™
Manuscripts

BMJ

I, the Submitting Author has the right to grant and does grant on behalf of all authors of the Work (as defined in the below author licence), an exclusive licence and/or a non-exclusive licence for contributions from authors who are: i) UK Crown employees; ii) where BMJ has agreed a CC-BY licence shall apply, and/or iii) in accordance with the terms applicable for US Federal Government officers or employees acting as part of their official duties; on a worldwide, perpetual, irrevocable, royalty-free basis to BMJ Publishing Group Ltd ("BMJ") its licensees and where the relevant Journal is co-owned by BMJ to the co-owners of the Journal, to publish the Work in this journal and any other BMJ products and to exploit all rights, as set out in our licence.

The Submitting Author accepts and understands that any supply made under these terms is made by BMJ to the Submitting Author unless you are acting as an employee on behalf of your employer or a postgraduate student of an affiliated institution which is paying any applicable article publishing charge ("APC") for Open Access articles. Where the Submitting Author wishes to make the Work available on an Open Access basis (and intends to pay the relevant APC), the terms of reuse of such Open Access shall be governed by a Creative Commons licence – details of these licences and which Creative Commons licence will apply to this Work are set out in our licence referred to above.

Other than as permitted in any relevant BMJ Author's Self Archiving Policies, I confirm this Work has not been accepted for publication elsewhere, is not being considered for publication elsewhere and does not duplicate material already published. I confirm all authors consent to publication of this Work and authorise the granting of this licence.

For peer review only

Validation of oxygen saturations measured in the community by emergency medical services as a marker of clinical deterioration in patients with confirmed COVID-19 - a retrospective cohort study

Matthew Inada-Kim[1]

Francis P. Chmiel[2]

Michael J. Boniface[2]

Daniel K. Burns[2]*

Helen Pocock[3,4]

John J. M. Black[3,5]

Charles D. Deakin[3,6]

[1] Acute Medical Unit, Department of Acute Medicine, Hampshire Hospitals NHS Foundation Trust, Winchester, United Kingdom.
[2] School of Electronics and Computer Science, University of Southampton, Southampton, SO17 1BJ, UK
[3] South Central Ambulance Service NHS Foundation Trust, Otterbourne, SO21 2RU, UK
[4] Warwick Clinical Trials Unit, University of Warwick, Coventry, CV4 7AL, UK
[5] Emergency Department, Oxford University Hospitals NHS Foundation Trust OX3 9DU.
[6] Southampton Respiratory Biomedical Research Unit, National Institute for Health Research, University Hospital Southampton, SO16 6YD

* Corresponding author: d.burns@soton.ac.uk

For peer review only - http://bmjopen.bmj.com/site/about/guidelines.xhtml

For peer review only

ABSTRACT

Objectives To evaluate oxygen saturation and vital signs measured in the community by emergency medical services (EMS) as clinical markers of COVID-19-positive patient deterioration.

Design A retrospective data analysis.

Setting Patients conveyed by EMS to two hospitals in Hampshire, UK between March 1st and July 31st 2020.

Participants A total of 1,080 patients aged >= 18 years old with a COVID-19 diagnosis who were conveyed by EMS to hospital.

Primary and secondary outcome measures The primary study outcome was admission to ICU within 30-days of conveyance with a secondary outcome representing mortality within 30-days of conveyance. ROC analysis was performed to evaluate, in a retrospective fashion, the efficacy of different variables in predicting patient outcomes.

Results Vital signs measured by EMS staff at first point of contact in the community correlated with patient 30-day ICU admission and mortality. Oxygen saturation was comparably predictive of 30-day ICU admission (AUROC 0.753 (95 % CI: 0.668-0.826)) to the NEWS2 score (AUROC 0.731 (95 % CI: 0.655-0.800), followed by temperature (AUROC 0.720 (95 % CI: 0.640-0.793)), and respiration rate (AUROC 0.672 (95 % CI: 0.586-0.756)).

Conclusions Initial oxygen saturation measurements (on air) for confirmed COVID-19 patients conveyed by EMS correlated with short-term patient outcomes, demonstrating an AUROC of 0.753 (95% CI: 0.668-0.826) in predicting 30-day ICU admission. We found that threshold of 93% Sp02 is prognostic of adverse events and of value for clinician decision making with sensitivity (74.2 % CI 0.642-0.840) and specificity (70.6 % CI 0.678-0.734).

==

For peer review only - http://bmjopen.bmj.com/site/about/guidelines.xhtml

ARTICLE SUMMARY

Strengths and limitations of this study

- We used baseline community oxygen saturation measurements (on air) for COVID-19 patients conveyed by emergency medical services (EMS) to hospital to evaluate efficacy of these measurements as prognostic factors for short-term (30-day) ICU admission and/or mortality.
- We also assessed the prognostic value of NEWS2 and other vital signs measured by EMS to provide contrast with our oxygen saturation results.
- The data is linked between EMS and hospital clinical records to enable our study.
- The data has limitations: only patients conveyed by emergency medical services were included, and the type of oxygen saturation measurement device for each patient was unknown.

For peer review only

INTRODUCTION

Severe acute respiratory syndrome coronavirus 2 (SARS-CoV-2) is a highly transmissible and pathogenic coronavirus that causes coronavirus disease 2019 (COVID-19) [1]. COVID-19 presents the biggest global healthcare challenge of our generation. As of February 2021, COVID-19 associated mortality stands at over 110,000 in the UK [2] COVID-19 presents a number of challenges in identifying optimal management pathways, not only in terms of the clinical care itself, but also identifying the stage at which hospital admission is necessary. Traditional management pathways involving paramedic assessment and conveyance to the Emergency Department (ED) for further review have proven impractical, not only because of the large numbers of patients involved, but because of the need to minimise contact of COVID-19 patients with others. Most patients who become symptomatic do so in a home environment where the majority will remain. In terms of optimising outcome, there is a need to understand which symptoms and signs in this environment are prognostic indicators of potential deterioration. The national recommendation for the implementation of COVID virtual wards recently announced by NHS England,[3] ushers in a novel approach of empowering patients through providing symptomatic, at risk patients a pulse oximeter and a toolkit for self-monitoring at home. It is hoped that this will enable the earlier recognition of deterioration in COVID-19 patients and potentially improved outcomes.

In most cases of bacterial and non-COVID pneumonia, breathlessness appears relatively early in the disease and ahead of any significant hypoxia. The challenge with assessing COVID-19 severity is that asymptomatic hypoxia often precedes breathlessness and by the time symptoms of breathlessness occur, patients have developed advanced disease and hypoxia may be significant.[4] The ability to detect this asymptomatic hypoxia before patients experience shortness of breath is critical for preventing respiratory involvement progressing to a life-threatening state. The key is to be able to detect this initial drop in oxygen saturation levels so that patients infected with COVID-19 who begin to suffer from pulmonary complications in the community can be detected early and conveyed to hospital for further treatment.[5] Although some studies have reported the relationship between oxygen saturation and outcome on presentation to the ED, we are not aware of any studies that have reported the relationship between oxygen saturations measured in the community by EMS and outcome. Patients who on assessment are severely hypoxic are clearly in need of emergency conveyance and hospital treatment, but by far the majority of patients with Covid-like symptoms seen and assessed by the EMS have relatively normal or near-normal oxygen saturations. These patients have generally not been conveyed and have been managed at home, but it has become apparent that even relatively minor derangements in oxygen saturations may be an early warning indicator for disease progression and the subsequent need for critical care. Use of oxygen saturation as an indicator

For peer review only

For peer review only - http://bmjopen.bmj.com/site/about/guidelines.xhtml

of disease severity may therefore underestimate the risk of leaving patients at home after assessment by the EMS. National case fatality rates (CFR) (ratio of deaths to total cases) have shown a strong inverse correlation between target oxygen saturation levels of 90-98% [6] suggesting that even mild derangements in oxygen saturation untreated can be detrimental to outcome.

Two small studies have suggested the utility of home oxygen monitoring for COVID-19 patients discharged from hospital,[7, 8] but no studies to our knowledge have used out-of-hospital oxygen saturation measurements as a trigger for initial hospital assessment. The purpose of this study therefore is to understand the prognostic significance of oxygen saturation when first measured by EMS clinicians. The understanding aims to inform escalation policies for safe and effective community-based triage and self-monitoring at home by identify a threshold where the sensitivity and specificity are of clinical value. It is hoped that the approach will contribute to hospital admission avoidance, enable earlier recognition of deterioration in COVID-19 patients and potentially improve outcome through early identification of those most at risk of disease progression. Whilst using a pulse oximeter provides a way for patients to monitor disease progression through a simple measurement procedure in contrast to the complexity of measurements required to calculate a NEWS2 score.

For peer review only

For peer review only - http://bmjopen.bmj.com/site/about/guidelines.xhtml

METHODS

Study Design

We undertook a retrospective review of clinically confirmed COVID-19 patients accessing a regional UK ambulance service who were conveyed to hospital and correlated their initial oxygen saturations measured at home with their in-hospital outcome. These were compared with the standard NEWS2 patient score, as used by all UK ambulance services, to identify the deteriorating patient.[9]

The cohort included adult patients (aged 18 years of older) initially assessed and conveyed by personnel from South Central Ambulance Service (SCAS) to the Emergency Department at one of the two hospitals within north Hampshire; Basingstoke & North Hampshire Hospital, or the Royal Hampshire County Hospital (Winchester) at which the patients were subsequently admitted.

The standard care pathway included 1) Patients calling emergency (999) and urgent (111) where they are triaged using NHS Pathways telephone script (release 19), 2) Attendance, assessment and monitoring by ambulance staff at the patient's home, 3) Conveyance to hospital for patients considered at high risk of deterioration 4) Admission to hospital and escalation to ICU for patients requiring critical care.

We analysed EMS conveyances occurring between 1st March to 31st July 2020, to determine suspect COVID-19 among conveyances at initial time of contact by the call taker or EMS staff, each patient record was reviewed for inclusion of at least one of the following four identifiers:

1. Those in who the EMS call taker had classified the call as 'COVID– Respiratory Distress'
2. Those where the Patient Clinical Record (PCR) listed the 'Presenting complaint' as 'Suspected COVID-19'.
3. Those where the PCR free text for the 'Presenting complaint' contained the word 'COVID'
4. Those where the PCR narrative in the free text field summarising the symptoms and their details completed by the paramedic contained the word 'COVID'.

Conveyances from these suspect COVID-19 patients were then linked to their subsequent hospital attendance. Of suspect cases, we then identified confirmed COVID-19 cases by selecting only those with a confirmed diagnosis in their discharge summary (i.e., the presence of a U07.1 or U07.2 ICD10 code). These confirmed COVID-19 cases made up our study cohort.

Seventeen patients did not have initial oxygen saturations recorded on air (but did have oxygen saturations recorded on oxygen) and were excluded from the data analysis. If this was because they were so obviously hypoxic clinically that EMS staff immediately administered oxygen without an initial

For peer review only - http://bmjopen.bmj.com/site/about/guidelines.xhtml

reading on air (or were constantly on home oxygen treatment), the ability of oxygen saturations to indicate risk of deterioration is likely to have been underestimated in this study.

All patients in known palliative care pathways were excluded from data analysis because their care did not follow standard care pathways.

Study setting

SCAS is a provider of emergency care in the counties of Hampshire, Berkshire, Buckinghamshire and Oxfordshire and covers a total of 3554 sq. miles (9205 km^2). The service receives approximately 500,000 emergency and urgent calls annually. SCAS covers a residential population of approximately 4.0 million inhabitants in a mix of urban and rural areas. The north Hampshire region forms part of the area covered by SCAS and comprises a residential population of approximately 306,000.[10]

Data collection

The initial oxygen saturation reading (SpO_2) on air recorded by the attending EMS staff (prior to any exercise or step test) and the NEWS2 score of patients fulfilling the inclusion criteria were collected from the EMS PCR. (NEWS2 score is calculated using the following seven variables: systolic blood pressure, heart rate, respiratory rate, temperature, oxygen saturation, supplemental oxygen administration, and level of consciousness - https://www.england.nhs.uk/ourwork/clinical-policy/sepsis/nationalearlywarningscore.)

Patient outcome was obtained by linking the SCAS and hospital clinical records by their NHS number. The primary outcome of our study was ICU admission within 30-days of conveyance and the secondary outcomes was mortality and a combined outcome (ICU admission and/or mortality) within 30-days of conveyance.

Data analysis

Analysis was performed in Python 3.7.2 [10], primarily making use of the statsmodels library [11]. Confidence intervals on observed mortality rates were estimated using the Wilson score interval. Where relevant, significance of the difference between two observed adverse outcome rates were tested using a two-population proportions z-test with the null hypothesis that the two-population proportions are equal.

For peer review only - http://bmjopen.bmj.com/site/about/guidelines.xhtml

To evaluate how predictive individual variables (e.g., oxygen saturation) and combinations of variables (e.g., oxygen saturation with age) were of 30-day adverse outcomes, we performed Receiving Operator Characteristics curve analysis. In the univariate analysis, we performed a complete case analysis (removing any patient with an incomplete record of vital signs) and assume a patient's adverse outcome risk is a linear function of the respective variable (where negative or positive correlation with outcome is assessed by clinical judgement) and calculated the ROC curve corresponding to if this variable alone was used to predict a patient's risk of an adverse outcome. We present both the sensitivity and specificity or the Area Under the Receiving Operator Characteristic curve (AUROC). The AUROC provides an estimate of the degree to which the predictor can discern between whether a patient has an adverse outcome within 30 days of conveyance or not, it can take values between 0.5 and 1.0. An AUROC of 0.5 corresponds to randomly guessing which patient have an adverse outcome within 30 days and an AUROC of 1.0 corresponds to a perfect classifier - it can predict, without error, who will have an adverse outcome within 30-days of conveyance. Confidence intervals were estimated by performing 1000 bootstrapping (sampling with replacement) iterations on the available data, calculating the AUROC on each of the samples and then calculating the relevant percentiles.

Patient and Public Involvement

This research was done without patient involvement. Patients were not invited to comment on the study design and were not consulted to develop patient-relevant outcomes or interpret the results. Patients were not invited to contribute to the writing or editing of this document for readability or accuracy.

For peer review only - http://bmjopen.bmj.com/site/about/guidelines.xhtml

RESULTS

A total of 19,868 patients were assessed at home and subsequently conveyed by EMS to North Hampshire Hospitals during the study period. The details of cohort selection are shown in Figure 1. The call handler or EMS staff identified 2,257 suspect COVID-19 cases and of these we identified 1,209 adults as having a confirmed diagnosis of COVID-19 (U07.1 or U07.2 coded in the patients discharge summary). Of the 1,209 confirmed cases we removed persons under palliative care (112 patients) and those with no initial oxygen saturation measurement on air recorded (17 patients). Overall, this left us with 1,080 confirmed COVID-19 patient records all of whom had initial oxygen saturation measurements on air. Of these 1,080, the complete records of vital signs were recorded at home by paramedics for 892 of the patients. The summary of the final patient cohort, with respect to demographics, comorbidities, and presence of vital sign measurements is given in Table 1. In our following discussions, we make use of all 1,080 patients, with the exception for our univariate analyses where we perform a complete case analysis and only use the 892 complete records.

Oxygen saturation was found to correlate with adverse outcome (Figure 2A), with lower initial oxygen saturation readings being associated with a higher mortality rate. In Figure 2A we display the correlation between the observed 30-day adverse outcome rates and initial oxygen saturation in detail, which displays correlation to all outcomes. In Table 2 we display the breakdown of our retrospective ROC analysis for using measured oxygen saturation as a binary triage tool (i.e., hospitalize or not) for different cut-offs (rows in Table 2). While the sensitivity vs specificity trade-off needs to be determined by the clinical context, this demonstrates that oxygen saturation is moderately discriminative for several cut-offs. For example, for a cut-off of 94 % or below, the sensitivity is 0.742 (95 % CI: 0.642-0.734) and the specificity is 0.706 (95 % CI: 0.678-0.734). Finally, we present comparisons of the results of ROC analysis for different variables measured in the community by EMS (Table 3). Across the three presented outcomes (30-day ICU admission, mortality, and combined outcome) correlations between variables and outcomes are broadly similar, with measured oxygen saturations and the NEWS2 score being the two most predictive of outcome. The notable differences are for the measured temperature which is moderately predictive of ICU admission (AUROC: 0.720 (95 % CI: 0.640-0.793)) but only weakly predictive of mortality (AUROC: 0.597 (95 % CI: 0.523-0.678)) and for patient age which is strongly positively correlated to mortality but displays a negative correlation to ICU admission (Figure 2B).

For peer review only - http://bmjopen.bmj.com/site/about/guidelines.xhtml

Variable	Outcome Category		
Outcome	No adverse event(n=955)	30-day ICU admission (n=58)	30-day mortality (n=78)
**Age**			
18-49	159 (16.6%)	11 (19.0%)	1 (1.3%)
50-59	132 (13.8%)	16 (27.6%)	2 (2.6%)
60-69	119 (12.5%)	17 (29.3%)	9 (11.5%)
70-79	209 (21.9%)	9 (15.5%)	16 (20.5%)
80+	336 (35.2%)	5 (8.6%)	50 (64.1%)
**Comorbidities**			
Chronic Obstructive Pulmonary Disorder	33 (3.5%)	0 (0.0%)	6 (7.7%)
Dementia	90 (9.4%)	1 (1.7%)	18 (23.1%)
Diabetes	216 (22.6%)	14 (24.1%)	14 (17.9%)
Kidney disease	7 (0.7%)	1 (1.7%)	3 (3.8%)
Chronic pain	37 (3.9%)	3 (5.2%)	1 (1.3%)
**Vital signs**			
Heart rate present	946 (99.1%)	58 (100.0%)	77 (98.7%)
Systolic blood pressure present	869 (91.0%)	51 (87.9%)	71 (91.0%)
Respiratory rate present	852 (89.2%)	49 (84.5%)	70 (89.7%)
Oxygen saturation (on air) present	955 (100.0%)	58 (100.0%)	78 (100.0%)
Temperature present	825 (86.4%)	49 (84.5%)	67 (85.9%)
ACVPU present	849 (88.9%)	50 (86.2%)	67 (85.9%)

Table 1: Characteristics of COVID-19 positive patients stratified by outcome. Note that n=11 patients experienced both ICU admission and mortality within 30 days. We only report on comorbidities which were present in the dataset as provided by the EMS. Comorbidity presence was recorded for every patient in the study. ACVPU = alert, confused, responding to voice, responding to pain, unresponsive. Oxygen saturations were not missing for any patients as those with missing values had been excluded (n=17). Overall, vital signs records were complete for 83% of cases.

For peer review only

For peer review only - http://bmjopen.bmj.com/site/about/guidelines.xhtml

Oxygen Saturation (on air) threshold (%)	Sensitivity (95 % CI)	Specificity (95 % CI)	Number of observations	Cumulative sum of number of observations
**85**	0.294 (0.200-0.400)	0.947 (0.933-0.962)	8	76
**86**	0.316 (0.216-0.421)	0.941 (0.927-0.955)	8	84
**87**	0.320 (0.216-0.432)	0.935 (0.920-0.950)	6	90
**88**	0.370 (0.261-0.476)	0.916 (0.899-0.933)	23	113
**89**	0.413 (0.304-0.523)	0.894 (0.874-0.913)	25	138
**90**	0.512 (0.411-0.615)	0.870 (0.849-0.890)	32	170
**91**	0.590 (0.477-0.699)	0.841 (0.823-0.867)	31	201
**92**	0.655 (0.544-0.761)	0.817 (0.796-0.841)	33	234
**93**	0.706 (0.593-0.803)	0.776 (0.751-0.801)	45	279
**94**	0.742 (0.642-0.840)	0.706 (0.678-0.734)	74	353
**95**	0.808 (0.718-0.892)	0.634 (0.605-0.662)	76	429
**96**	0.848 (0.767-0.921)	0.508 (0.477-0.538)	129	558
**97**	0.898 (0.822-0.963)	0.357 (0.330-0.386)	156	714
**98**	0.911 (0.841-0.973)	0.226 (0.201-0.254)	132	846
**99**	0.961 (0.913-1.0)	0.091 (0.075-0.109)	139	985
**100**	1	0	95	1080

Table 2: Evaluation of initial oxygen saturation measured by paramedics in COVID-19 patients in the community used as a binary classifier for predicting 30-day ICU admission within 30 days of conveyance. Each row denotes a different threshold for determining those at risk of an adverse outcome. We display the sensitivity and specificity for each threshold, equivalent to all possible intersections of the receiving operator curve using thresholds between 85 % and 100 %. In total 68 patients had an oxygen saturation of 84 % or less (not shown). The column on the far right denotes the cumulative sum of the number of observations of the given oxygen saturation (row) or below. For example, 76 patients had an oxygen saturation of 85 % or less recorded (top row) and 429 patients had an oxygen saturation of 95 % or less recorded. Confidence intervals are estimated by bootstrapping.

For peer review only

	AUROC (95 % CI)		
Variable	ICU admission	Mortality	Combined
**Oxygen Saturation (on air)**	0.753 (0.668-0.826)	0.778 (0.704-0.843)	0.775 (0.727-0.829)
**NEWS2**	0.731 (0.655-0.800)	0.768 (0.709-0.823)	0.760 (0.708-0.807)
**Respiration rate**	0.672 (0.586-0.756)	0.668 (0.599-0.736)	0.677 (0.618-0.738)
**Temperature**	0.720 (0.640-0.793)	0.597 (0.523-0.678)	0.636 (0.69-0.700)
**Systolic blood pressure**	0.634 (0.560-0.706)	0.604 (0.529-0.680)	0.626 (0.568-0.684)
**Heart rate**	0.590 (0.506-0.672)	0.558 (0.486-0.631)	0.574 (0.514-0.633)
**Age band**	0.670 (0.611-0.734)	0.685 (0.626-0.738)	0.557 (0.495-0.615)

Table 3: Ranked Area Under Receiver Operator Curves (AUROC) calculated for isolated physiological variables and the composite NEWS2 score with each outcome. AUROCS were calculated using a complete case analysis with 892 patients in total. Confidence intervals are estimated by bootstrapping, with 95 % confidence intervals presented alongside the mean validation AUROC across samples.

For peer review only

For peer review only - http://bmjopen.bmj.com/site/about/guidelines.xhtml

For peer review only

For peer review only - http://bmjopen.bmj.com/site/about/guidelines.xhtml

DISCUSSION

Community assessment of patients with COVID-19 symptoms using a single initial oxygen saturation on air measurement correlates with 30-day clinical outcomes. Qualitatively, the observed 30-day adverse outcome rate is approximately constant between oxygen saturations of 100 - 96 % and then increase with decreasing oxygen saturation from 95 % to 90 %. Below 90 %, the mortality risk remains high. Although the therapeutic target range for oxygen saturations in the UK is 94-98%,[11] and in the USA is 92-96%,[12] this study suggests that patients at the lower end of this range are still at risk of deterioration in the context of COVID-like symptoms. For example, for patients in our cohort with presenting oxygen saturations in the range of 92-94 %, values often regarded as within this normal range, had a significantly ($p=0.025$) higher risk of ICU admission within 30 days (5.9 %) compared to those presenting with oxygen saturations greater than 95 % (ICU admission rate 2.5 %). Outside this 'normal' range, our analysis suggests even relatively small decreases in oxygen saturation are markers of increased risk of death or ICU admission and suggest that a lower threshold for hospital conveyance may be necessary for patients who traditionally would be considered to have only minor physiological derangement and otherwise have been left at home.

The sensitivity of home oxygen saturation measurements reflects the percentage of people correctly identified with adverse outcomes. The sensitivity of this parameter for adverse outcome decreased as oxygen saturation fell (Table 2). An oxygen saturation ≤ 90% was associated with a relatively low sensitivity of < 0.5. Specificity of identifying an adverse outcome, an indirect measure of unnecessary conveyance to hospital (but also including patients who survived and did not need ICU admissions), increased as oxygen saturations fell. However, it is important to ensure that patients at risk of deterioration are not missed and a degree of over-triage would be necessary to ensure that this was not the case. However, even oxygen saturations at the lower end of the normal range are associated with a risk of deterioration (sensitivity of 94% saturations = 0.713) and it therefore appears that oxygen saturation alone has significant limitations when it is within a normal range.

Although oxygen saturations as a risk factor for COVID-19 patients on presentation to the Emergency Department are widely reported,[13, 14] the ability of oxygen saturations measured in the community to indicate disease severity and the need for hospital conveyance has not been widely reported, presumably because of the challenges in equipping patients with pulse oximeters prior to the onset of any illness. Several studies have used oxygen levels in patients presenting in the ED as an indicator of the need for hospital admission and others have used the opportunity to send ED patients not requiring admission home with a pulse oximeter for self-monitoring. Oxygen saturations on presentation to the ED have also been shown to be strongly associated with outcome. The strongest

For peer review only - http://bmjopen.bmj.com/site/about/guidelines.xhtml

critical illness risk has been shown to be admission oxygen saturation < 88% (OR 6.99).[14] Other studies have shown that even a relatively mildly deranged oxygen saturation of <92% is strongly associated with an increased risk of in-hospital mortality.[15] Conversely, an ED resting $SpO_2 \geq 92\%$ as part of discharge criteria can achieve hospital readmission rates as low as 4.6%, [16] suggesting that it may be a safe threshold for discharge in symptomatic patients with mild disease after diagnostic workup .

Home oxygen saturation monitoring has been used for patients discharged from hospital, either from the ED because their disease was not severe, or from intensive care for convalescence. A small study of patients with COVID-19 discharged from an ED, reported similar results to ours using subsequent home oxygen saturation monitoring. In these patients, resting home $SpO_2 < 92\%$ was associated with an increased likelihood of re-hospitalization compared to $SpO_2 \geq 92\%$ (relative risk = 7.0, 95% CI 3.4 to 14.5, $p < 0.0001$). Home $SpO_2 < 92\%$ was also associated with increased risk of intensive care unit admission.[8]

Oxygen saturation is an integral variable in most critical illness tools. The association of prehospital oxygen saturation has been shown to be predictive of 2-day mortality [17]and has been used to identify COVID-19 patients requiring hospital admission.[18] NHS England has encouraged the use of the NEWS2 scoring system to identify patients at risk of deterioration. This uses weighted physiological variables of heart rate, systolic blood pressure, oxygen saturation (on air), respiratory rate, temperature and level of consciousness to produce a score that is correlated with risk of deterioration, not only as a general illness score, but specifically in patients with known COVID-19.[19] NEWS2 has been compared with a quick COVID Sensitivity Index (qCSI), a test that includes SpO2, respiratory rate and O2 flow rate to calculate a score between 1 and 12, and risk level. The study concludes NEWS2 is significantly better than qCSI, with AUC of 0.779 and 0.750 respectively [20]. Furthermore, qCSI does not consider severity score for readings of 93% and above, whilst qCSI pulse oximetry readings are the lowest reading recorded during the first 4 hours of patient encounter at the hospital, rather than being prior to admission. In our study, we were concerned with the ability of isolated oxygen saturations measured by EMS on attendance in comparison with NEWS2 in our cohort to identify patients at risk of ICU admission (and mortality) within 30 days. Using ROC analysis, the AUROC for oxygen saturations at predicting ICU admission alone was 0.753 (95% CI 0.668-0.826) and for NEWS2 was 0.731 (95% CI 0.655-0.800). These results are consistent with a previous study using NEWS2 scores on hospital admission which has shown an AUROC of 0.822 (95% CI 0.690-0.953) to predict risk of severe disease.[19] The lower observed AUROC of NEWS2 compared to oxygen saturations may be the result of the NEWS2 score incorporating physiological variables less predictive of COVID-19 outcomes than

For peer review only - http://bmjopen.bmj.com/site/about/guidelines.xhtml

oxygen saturations, thereby reducing the discriminative ability of the score, or because it uses discretized oxygen saturations which amounts to information loss. Additionally, we have not assessed the reporting compliance of the NEWS2 scores and this may have impacted the observed AUROCs. Interestingly, a recent review of 22 prognostic models showed that oxygen saturation on room air and patient age were strong predictors of deterioration and mortality among hospitalised adults with COVID-19 respectively, but no other variables added incremental value to these predictors.[18] We have shown the same for oxygen saturation as a univariate predictor in the pre-hospital setting, and that predictive value does not increase by the addition of other physiological variables. The PRIEST study using NEWS2, age, sex, and performance status of patients in the ED predicted adverse outcome with good discrimination in adults with suspected COVID-19 [20]. The discriminatory ability of this more complex scoring system was similar to that demonstrated by simply measuring the oxygen saturations in the community and further reinforces the utility of home oxygen saturations as a simple marker, not only for use by the EMS, but by members of the public equipped with home oximetry.

A number of remote home monitoring models for patients with suspected COVID-19 have been proposed, all of which aim to achieve early identification of deterioration for patients self-managing COVID-19 symptoms at home.[21] It would be expected that the utility of home monitoring would be improved by the ability to measure oxygen saturations, although not all models currently integrate this into their protocols. Our results show that resting oxygen saturations measured in patients with confirmed COVID-19 perform on a par with the same measurements taken in the ED. They therefore suggest that the predictive value of oxygen saturations may be able to be effectively moved to an earlier stage in the disease process and measured while the patient is still at home. Although initial home SpO_2 may provide a useful marker of disease severity and the need for hospital conveyance, it is clear that it has limited sensitivity and may need to be interpreted as part of an overall assessment of the patient. Some authors have argued that pulse oximetry identified the need for hospitalisation when using a cut- off of 92%,[8] but based on our data (Table 2), approximately one-third of patients with an adverse outcome would be missed using this threshold. We have demonstrated that even patients presenting with oxygen saturations of 92-94 %, which are values often regarded as within a normal range, have a higher mortality than those with oxygen saturations higher than 95 %. Even when measured in the ED, baseline median SpO_2 was as high as 95.0 % in those with an adverse outcome, compared to 97.0% in those without.[22] It is clear that the relatively low sensitivity of oxygen saturation in those with mildly deranged values limits the utility of this parameter alone in assessing risk of adverse outcome.

For peer review only - http://bmjopen.bmj.com/site/about/guidelines.xhtml

This is a relatively small retrospective cohort study with concomitant limitations of sample size. The subjective nature of paramedic classification of symptoms consistent with COVID-19 may have introduced some degree of bias into patients included in the study, as may have the presence of known co-morbidities. Our dataset did not include patients who were reviewed by EMS but not conveyed to hospital and this is arguably the most significant source of bias in our study. It is reasonable that for patients where a decision was made not to convey them, they were less likely to deteriorate and more likely to have normal vital signs. If this is the case, this would result in a reduction of the discriminative ability of recorded oxygen saturations. We did not specifically compare the outcome data of COVID and Non-COVID patients with mildly deranged oxygen saturations. However, our data suggests that mild derangement in COVID patients is a significant risk factor for deterioration and this does not match the clinical progression witnessed in non-COVID patients. We acknowledge that for very low Sp02 levels our results show poor clinical value and we believe this is due to other factors influencing escalation decisions that are not included in our dataset. Patients on palliative care pathways were also removed from the study cohort, but are likely to be more susceptible to deterioration from COVID, irrespective of any alternative care pathway.

With waves of COVID-19 regularly overwhelming EMS and hospital services, there is an urgent need to optimise the identification of patients at risk of deterioration. We undertook this research to ascertain the role simple physiological measures might have to inform clinical decision making. While the results are hypothesis-forming (i.e., it shows oxygen saturations are predictive of clinical outcomes within the care pathway studied in this manuscript), it has clinical utility as it helps inform decisions made by clinicians at the point of conveyance. This will enable more patients to be safely managed in the community and only referred to hospital once their clinical symptoms and physiological signs suggest a risk of deterioration and the need for hospital care. This is particularly needed for the majority of patients who have mild to moderate symptoms where it is not clear if community or hospital management is appropriate. Home pulse oximetry is becoming relatively cheap and easily accessible for the public and may be a relatively cost-effective tool in the safe community management of these patients, perhaps focussed on those with significant co-morbidities who are at higher risk. The utility of remote monitoring systems (or the COVID virtual ward) has been an increasingly studied subject, and there is growing evidence that remote monitoring can facilitate more streamlined approaches to the delivery of patient care, especially in pulmonary disease.[7] The use of ICU admission as an endpoint identifies patients seen at home who go on to deteriorate and the correlation of home oxygen saturation with a risk of severe deterioration assists ambulance crews in identifying both those who should be conveyed to hospital as well as those who can, with a reasonable

For peer review only - http://bmjopen.bmj.com/site/about/guidelines.xhtml

degree of certainty, be safely left at home. Further prospective studies are required to understand the utility of home pulse oximetry, but this study suggests that it may have the potential to significantly contribute to the safe and appropriate management of these patients in the community with timely referral to hospital when indicated.

Conclusions

We have demonstrated that even relatively minor derangements in peripheral oxygen saturation are an early warning of potential deterioration in confirmed COVID-19 patients conveyed by EMS to hospital and oxygen saturation would appear to have potential to be a key physiological variable that together with other clinical signs and clinical risk factors may be able to identify patients at risk of deterioration.

Acknowledgements

We thank Simon Mortimore and Philip King from South Central Ambulance Service and Zoe Cameron from Hampshire Hospitals NHS Foundation Trust for their assistance in data extraction and analysis.

Funding statement

This report includes independent research funded by the National Institute for Health Research Applied Research Collaboration Wessex. The views expressed in this publication are those of the author(s) and not necessarily those of the National Institute for Health Research or the Department of Health and Social Care.

Governance and ethics approval statement

Regulatory and ethical approval for the study were provided by the Health Research Authority (REC reference 20/HRA/5445) and by the University of Southampton Ethics Committee (REF ERGO/61242). NHS England and NHS Improvement have been given legal notice by the Secretary of State for Health and Social Care to support the processing and sharing of information to help the COVID-19 response under Health Service Control of Patient Information Regulations 2002 (COPI). This is to ensure that confidential patient information can be used and shared appropriately and lawfully for purposes related to the COVID-19 response. Data were extracted from medical records by clinicians providing care for the patients and an anonymised extract of the data were provided to the team at the University of Southampton.

Author contributions

For peer review only

For peer review only - http://bmjopen.bmj.com/site/about/guidelines.xhtml

MI-K, MJB, JJM Black, CDD led and conceptualized the study. MI-K led at HHFT, MJB led at UoS, CDD led at SCAS. FPC and DKB performed the data analysis with support and guidance from all authors. MI-K, HP and JJM Black performed the data extraction. MJB led the data governance. CDD and HP provided clinical insight. MI-K, CDD, HP and FPC wrote the first draft of the manuscript. All authors discussed the results. All others contributed to subsequent drafts of the manuscript. DKB prepared the final manuscript for submission.

Competing interests

M. I-K. is National Clinical Lead Deterioration & National Specialist Advisor Sepsis, NHS England and NHS Improvement. All other authors declare no competing interests.

Data accessibility

Due to information governance concerns, the data will not be made public. However, it will be made accessible via reasonable request to the corresponding author.

For peer review only

References

1. Hu, B., et al., *Characteristics of SARS-CoV-2 and COVID-19.* Nature Reviews Microbiology, 2021. **19**(3): p. 141-154.
2. England, P.H. *Coronavirus (COVID-19) in the UK*. Available from: https://coronavirus.data.gov.uk.
3. *Pulse oximetry to detect early deterioration of patients with COVID-19 in primary and community care settings, 11 June 2020, Version 1, NHS.* *https://www.england.nhs.uk/coronavirus/publication/pulse-oximetry-to-detect-early-deterioration-of-patients-with-covid-19-in-primary-and-community-care-settings Accessed 28 Oct 2020.*
4. Bickler, P.E., et al., *"Silent" Presentation of Hypoxemia and Cardiorespiratory Compensation in COVID-19.* Anesthesiology, 2020.
5. Teo, J., *Early Detection of Silent Hypoxia in Covid-19 Pneumonia Using Smartphone Pulse Oximetry.* J Med Syst, 2020. **44**(8): p. 134.
6. Goyal, D., Donnelly H., Kussner, A., Bhatti, N.J., Mansab, F., *Oxygen and mortality in COVID-19 pneumonia: a comparative analysis of supplemental oxygen policies and health outcomes across 26 countries.* 2020.
7. O'Carroll, O., et al., *Remote monitoring of oxygen saturation in individuals with COVID-19 pneumonia.* Eur Respir J, 2020. **56**(2).
8. Shah, S., et al., *Novel use of home pulse oximetry monitoring in COVID-19 patients discharged from the emergency department identifies need for hospitalization.* Acad Emerg Med, 2020.
9. *Royal College of Physicians National Early Warning Score (NEWS) 2. London: RCP, 2017. www.rcplondon.ac.uk/projects/outputs/national-early-warning-score-news-2 [Accessed 19 Oct 2020].*
10. *https://www.hants.gov.uk/landplanningandenvironment/facts-figures/population/estimates-forecasts. Accessed 17th October 2020.*
11. O'Driscoll, B.R., et al., *BTS guideline for oxygen use in adults in healthcare and emergency settings.* Thorax, 2017. **72**(Suppl 1): p. ii1-ii90.
12. *COVID-19 Treatment Guidelines Panel. Coronavirus Disease 2019 (COVID-19) Treatment Guidelines. National Institutes of Health. Available at https://www.covid19treatmentguidelines.nih.gov/. Accessed 15th October 2020.*
13. Gidari, A., et al., *Predictive value of National Early Warning Score 2 (NEWS2) for intensive care unit admission in patients with SARS-CoV-2 infection.* Infect Dis (Lond), 2020. **52**(10): p. 698-704.
14. Petrilli, C.M., et al., *Factors associated with hospital admission and critical illness among 5279 people with coronavirus disease 2019 in New York City: prospective cohort study.* BMJ, 2020. **369**: p. m1966.
15. Mikami, T., et al., *Risk Factors for Mortality in Patients with COVID-19 in New York City.* J Gen Intern Med, 2020.
16. Berdahl, C.T., et al., *The safety of home discharge for low-risk emergency department patients presenting with coronavirus-like symptoms during the COVID-19 pandemic: A retrospective cohort study.* JACEPOPEN, 2020. **August 2020. https://doi.org/10.1002/emp2.12230**.
17. Martín-Rodríguez, F., et al., *Association of Prehospital Oxygen Saturation to Inspired Oxygen Ratio With 1-, 2-, and 7-Day Mortality.* JAMA Network Open, 2021. **4**(4): p. e215700-e215700.
18. Gupta, R.K., et al., *Systematic evaluation and external validation of 22 prognostic models among hospitalised adults with COVID-19: An observational cohort study.* Eur Respir J, 2020.

For peer review only

For peer review only - http://bmjopen.bmj.com/site/about/guidelines.xhtml

19. Myrstad, M., et al., *National Early Warning Score 2 (NEWS2) on admission predicts severe disease and in-hospital mortality from Covid-19 - a prospective cohort study.* Scand J Trauma Resusc Emerg Med, 2020. **28**(1): p. 66.
20. Martín-Rodríguez, F., et al., *One-on-one comparison between qCSI and NEWS scores for mortality risk assessment in patients with COVID-19.* Annals of Medicine, 2022. **54**(1): p. 646-654.
21. Vindrola-Padros, C., et al., *Remote home monitoring (virtual wards) during the COVID-19 pandemic: a living systematic review. 2020. https://www.medrxiv.org/content/10.1101/2020.10.07.20208587v1.full.pdf.*
22. *Goodacre, S. , Thomas, B., Lee, E. et al. Post-exertion oxygen saturation as a prognostic factor for adverse outcome in patients attending the emergency department with suspected COVID-19 : observational cohort study. medRxiv. (Submitted:2020).*

For peer review only

For peer review only

For peer review only - http://bmjopen.bmj.com/site/about/guidelines.xhtml

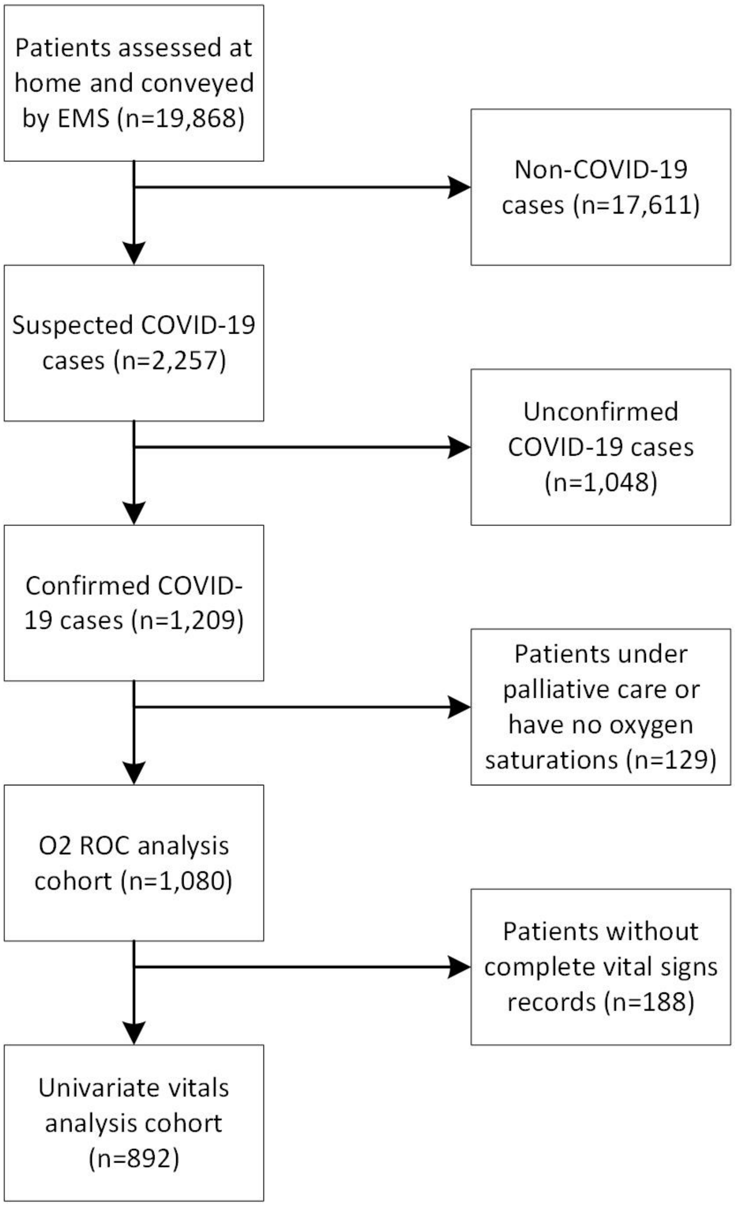

The cohort selection of the EMS patients.

71x115mm (300 x 300 DPI)

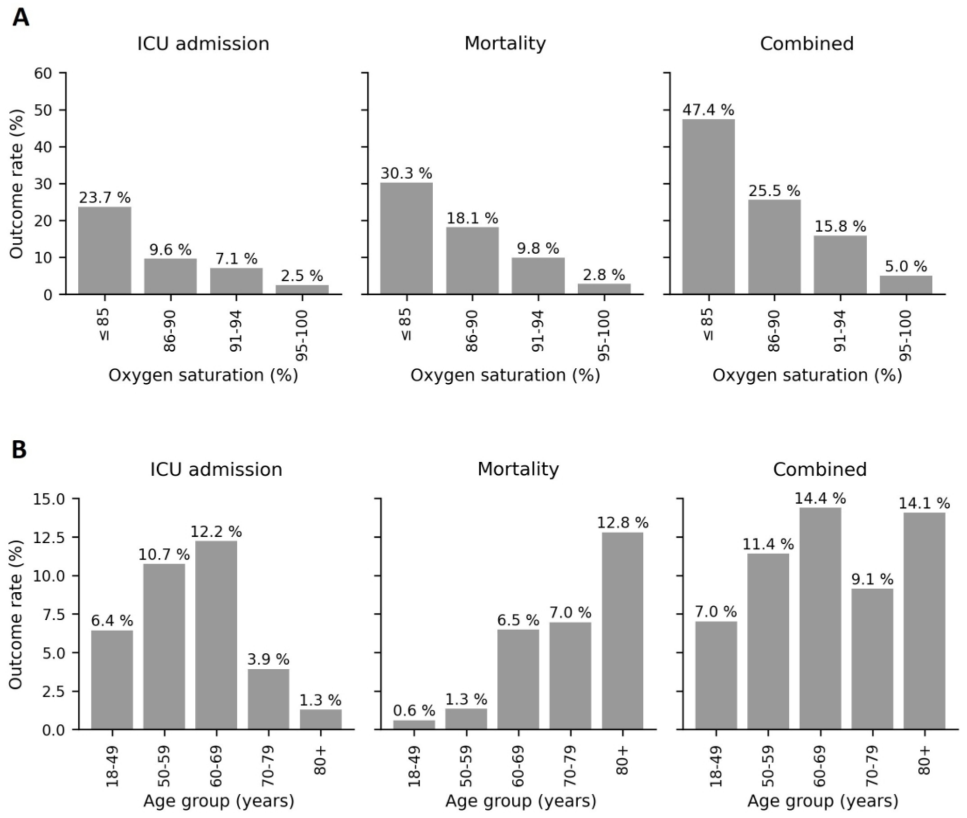

A) The ICU admission, mortality, and combined outcome rates as a function of oxygen saturation %. B) The ICU admission, mortality, and combined outcome rates as a function of age group.

188x166mm (300 x 300 DPI)

STROBE Statement—Checklist of items that should be included in reports of ***cohort studies***

	Item No	Recommendation	Page No
**Title and abstract**	1	(*a*) Indicate the study's design with a commonly used term in the title or the abstract	1
		(*b*) Provide in the abstract an informative and balanced summary of what was done and what was found	2
**Introduction**			
Background/rationale	2	Explain the scientific background and rationale for the investigation being reported	5
Objectives	3	State specific objectives, including any prespecified hypotheses	6
**Methods**			
Study design	4	Present key elements of study design early in the paper	7
Setting	5	Describe the setting, locations, and relevant dates, including periods of recruitment, exposure, follow-up, and data collection	7
Participants	6	(*a*) Give the eligibility criteria, and the sources and methods of selection of participants. Describe methods of follow-up<br>(*b*) For matched studies, give matching criteria and number of exposed and unexposed	7, 8
Variables	7	Clearly define all outcomes, exposures, predictors, potential confounders, and effect modifiers. Give diagnostic criteria, if applicable	8, 9
Data sources/ measurement	8*	For each variable of interest, give sources of data and details of methods of assessment (measurement). Describe comparability of assessment methods if there is more than one group	8, 9
Bias	9	Describe any efforts to address potential sources of bias	16
Study size	10	Explain how the study size was arrived at	N/A
Quantitative variables	11	Explain how quantitative variables were handled in the analyses. If applicable, describe which groupings were chosen and why	8, 9
Statistical methods	12	(*a*) Describe all statistical methods, including those used to control for confounding<br>(*b*) Describe any methods used to examine subgroups and interactions<br>(*c*) Explain how missing data were addressed<br>(*d*) If applicable, explain how loss to follow-up was addressed<br>(*e*) Describe any sensitivity analyses	8, 9
**Results**			
Participants	13*	(a) Report numbers of individuals at each stage of study—eg numbers potentially eligible, examined for eligibility, confirmed eligible, included in the study, completing follow-up, and analysed<br>(b) Give reasons for non-participation at each stage<br>(c) Consider use of a flow diagram	7, 10
Descriptive data	14*	(a) Give characteristics of study participants (eg demographic, clinical, social) and information on exposures and potential confounders	Figures 1, 2 Table 1
		(b) Indicate number of participants with missing data for each variable of interest	Pages 11, 12, 13
		(c) Summarise follow-up time (eg, average and total amount)	

For peer review only

Outcome data	15*	Report numbers of outcome events or summary measures over time	10
Main results	16	(*a*) Give unadjusted estimates and, if applicable, confounder-adjusted estimates and their precision (eg, 95% confidence interval). Make clear which confounders were adjusted for and why they were included<br>(*b*) Report category boundaries when continuous variables were categorized<br>(*c*) If relevant, consider translating estimates of relative risk into absolute risk for a meaningful time period	10
Other analyses	17	Report other analyses done—eg analyses of subgroups and interactions, and sensitivity analyses	11, 12
**Discussion**			
Key results	18	Summarise key results with reference to study objectives	14, 15
Limitations	19	Discuss limitations of the study, taking into account sources of potential bias or imprecision. Discuss both direction and magnitude of any potential bias	16, 17
Interpretation	20	Give a cautious overall interpretation of results considering objectives, limitations, multiplicity of analyses, results from similar studies, and other relevant evidence	17, 18
Generalisability	21	Discuss the generalisability (external validity) of the study results	16, 17
**Other information**			
Funding	22	Give the source of funding and the role of the funders for the present study and, if applicable, for the original study on which the present article is based	19

*Give information separately for exposed and unexposed groups.

Note: An Explanation and Elaboration article discusses each checklist item and gives methodological background and published examples of transparent reporting. The STROBE checklist is best used in conjunction with this article (freely available on the Web sites of PLoS Medicine at http://www.plosmedicine.org/, Annals of Internal Medicine at http://www.annals.org/, and Epidemiology at http://www.epidem.com/). Information on the STROBE Initiative is available at http://www.strobe-statement.org.

For peer review only
